# Automated spermatogenic staging in periodic acid-Schiff-stained testes of Sprague–Dawley rats using a deep learning model for normal and atrophied tissues

**DOI:** 10.1371/journal.pone.0337245

**Published:** 2026-06-29

**Authors:** Da-Mi Kim, Jin-Hyung Rho, So-Young Wee, Hwa-Young Son

**Affiliations:** 1 Department of Veterinary Medicine, Chungnam National University, Daejeon, Republic of Korea; 2 Pathology Team, Corestemchemon Nonclinical Research Institute, Yongin, Republic of Korea; 3 Jeonbuk Pathology Research Group, Korea Institute of Toxicology, Jeongeup, Republic of Korea; University of Hyderabad, INDIA

## Abstract

The spermatogenic stage serves as a vital criterion for assessing normal spermatogenesis and is central to evaluating reproductive toxicity. Current manual methods for evaluating the spermatogenic stage are time-intensive, require expert knowledge, and are less effective at detecting subtle changes or comparing stage frequencies across samples. To overcome these limitations, this study introduces a method that leverages object detection models and Region-based Convolutional Neural Networks to enable efficient and accurate evaluation of spermatogenic stages. A total of 16 periodic acid-Schiff-stained testicular tissue whole-slide images (WSIs) obtained from 16 Sprague–Dawley rats were used in this study. A total of 14 stages were identified, and the approach was further applied to atrophied testicular samples as a real-world example. A total of 10 WSIs (nine normal and one atrophied testes) were used for model training, validation, and testing. Six additional WSIs (three normal and three atrophied testes) were used for model inference. For the test set, the model achieved a mean average precision of 0.869 and a mean average recall of 0.977 for detecting spermatogenic stages and atrophy. For the inference set, agreement with pathologist assessments exceeded 91%, providing objective benchmarks for stage evaluation and facilitating the comparison of stage frequencies across multiple samples. The model enabled the quantitative assessment of atrophied tissues by analyzing the proportional changes in atrophied seminiferous tubules relative to normal tubules. This automated approach has the potential to reduce the workload of pathologists by enabling rapid, reproducible assessment of toxicological changes during spermatogenesis. As a proof-of-concept, the integration of deep learning demonstrated the feasibility of improving the efficiency and objectivity of pathological evaluations in reproductive toxicity studies.

## Introduction

Spermatogenic staging is a critical evaluation criterion in nonclinical reproductive toxicity studies, offering an indirect assessment of whether spermatogenesis is proceeding normally. Spermatogenesis is the process by which spermatogonia undergo mitotic and meiotic division to produce spermatozoa, which are eventually released into the lumen [1]. In rats, this process is classified into 14 distinct stages based on the progression of the seminiferous epithelium [2]. The periodic acid-Schiff staining(PAS) enables the observation of 14 stages by highlighting polysaccharides within the acrosome of spermatids [3], whereas only eight stages were observed in hematoxylin and eosin (H&E)-stained slides.

Testicular atrophy is one of the important lesions indicative of reproductive toxicity. The pathogenesis of seminiferous tubule atrophy involves damage to Sertoli cells, cytotoxicity, hypoxia, and inflammation, which can lead to germ cell depletion and, in severe cases, a reduction in testis size or weight [4]. Since seminiferous tubule atrophy can occur spontaneously [5], for a toxicologic pathologist, it is important to quantify the number of atrophied tubules and assign an appropriate severity grade for distinguishing treatment-related toxicity from spontaneous background lesions. The Organization for Economic Cooperation and Development guidelines emphasize histopathological evaluation as the most sensitive tool for identifying adverse effects on male reproductive function [6,7]. However, manual evaluation is not only labor-intensive but also inherently subjective, leading to potential inter-pathologist variability. In practice, only a limited portion of the testis is evaluated, which may fail to detect subtle, stage-related abnormalities or shifts in stage distributions. To address this issue, previous studies have demonstrated several potential deep learning models to automate spermatogenic stage evaluation in laboratory animals, including mice, rats, dogs, and nonhuman primates, thereby reducing human workload while enabling quantitative, reproducible analysis. To automate this process, previous approaches have relied on U-Net–based segmentation pipelines applied to H&E-stained whole-slide images (WSIs). In these workflows, individual seminiferous tubules are first segmented, followed by germ cell segmentation within each tubule, and spermatogenic stages are subsequently inferred using rule-based or decision tree classifiers derived from germ cell morphology [8–11]. Although these segmentation-based methods achieved high accuracy in localized tasks, they present several practical limitations. First, segmentation requires pixel-level processing, which is computationally intensive and time-consuming, particularly for WSI-scale inference. Second, the multi-step nature of these pipelines introduces error propagation, whereby inaccuracies in tubule segmentation adversely affect downstream germ cell analysis and final stage classification. Third, because most prior studies relied on H&E staining, which does not clearly visualize the acrosome, full discrimination of all 14 spermatogenic stages was not feasible. As a result, several stages were grouped into broader categories, limiting biological resolution and interpretability.In this study, we introduce a two-stage detector–based object detection framework for spermatogenic stage classification using PAS-stained histological images to address previous limitations and improve practical applicability in toxicologic pathology. Implementing two-stage object detection, which belongs to Region-based Convolutional Neural Network (R-CNN), substantially reduced computational overhead and inference time and gained high accuracy. We also compared the performance of the model with two widely used pretrained models in the R-CNN family – Faster R-CNN [12] and Cascade R-CNN [13]. Additionally, training PAS-stained testis enabled accurate 14 spermatogenic stages, making it more suitable for large-scale toxicity studies involving a large number of WSIs. As a practical demonstration, the proposed model was evaluated on three normal testes and three atrophied testes to quantify stage distributions and atrophied lesions. In addition to the quantification, the atrophied testes were classified into three grades based on the results.

## Materials and methods

### Animal ethics

This study involved the secondary use of animal images obtained in a previously approved study. The original animal experiments were approved by the Institutional Animal Care and Use Committee (IACUC) of Corestemchemon Inc. (approval number: 21-R603). No additional animal experiments were performed, and the use of images alone did not require additional IACUC approval.

### Preparation of the testis sample

Histopathological images used in this study were obtained from a previous animal study. No additional animal experiments were performed for the purposes of this study.

In the original study, the animals were anesthetized with isoflurane delivered via inhalation until a deep surgical plane of anesthesia was achieved, as confirmed by the complete loss of pedal withdrawal and corneal reflexes. Under deep anesthesia, the animals were euthanized via transection of the abdominal aorta and inferior vena cava. This was performed as a terminal procedure to ensure rapid death and minimize pain and distress. All efforts were made to alleviate animal suffering, and no animals regained consciousness before euthanasia.

### Image processing and annotation

#### Digital image processing.

A total of 16 WSIs were obtained from 16 rats. Corestemchemon Inc. (Yongtin, Republic of Korea) provided 16 PAS-stained testis slides of 21-week-old Sprague–Dawley rats, consisting of 12 normal and 4 atrophied testes. Animals were acquired from Orient Bio Inc., and the PAS stain kit was purchased from ScyTek Laboratories Inc (West Logan, UT, USA). Transverse sections of the right testis were obtained. A total of 16 slides from 16 animals were scanned at 40 × magnification using a 3DHistech Ltd. (Budapest, Hungary) slide scanner. A total of 10 digitized WSIs (nine normal testes and one atrophied testis) were cropped into 4096 × 4096 × 3 resolution tiles using the OpenSlide library [14] to facilitate deep learning model labeling for training. The remaining six WSIs (three normal testes and three atrophied testes) were reserved for inference.

#### Image annotation.

**Fig 1:**
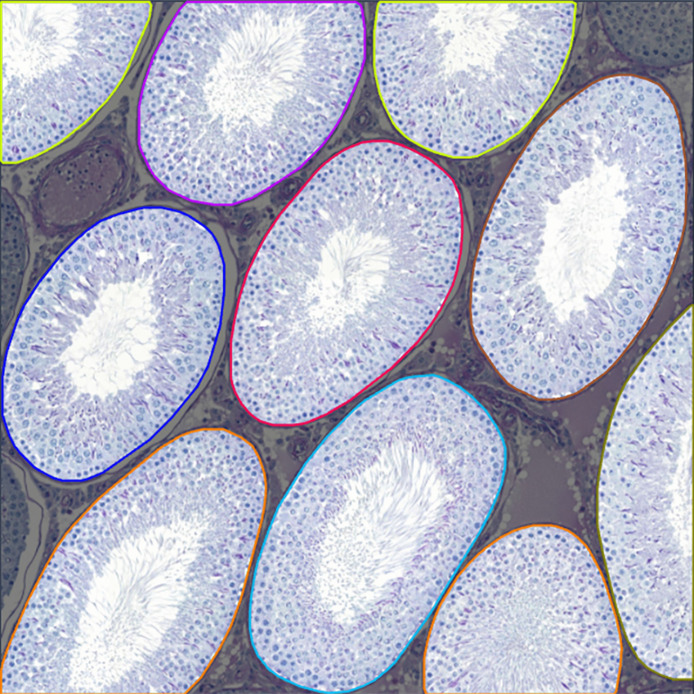
One example of class labeling on a tile image. Each class was distinguished by a specific color. The spermatogenic stages according to color were as follows —yellow green: stage I, red: stage II–III, purple: stage V, orange: stage VI, sky blue: stage VII, blue: stage XII, brown: stage XIII, khaki: stage XIV.

A total of 15,649 seminiferous tubules were annotated from 683 tile images using the Roboflow software (Roboflow, Des Moines, IA, USA) as shown in Fig 1.

**Fig 2:**
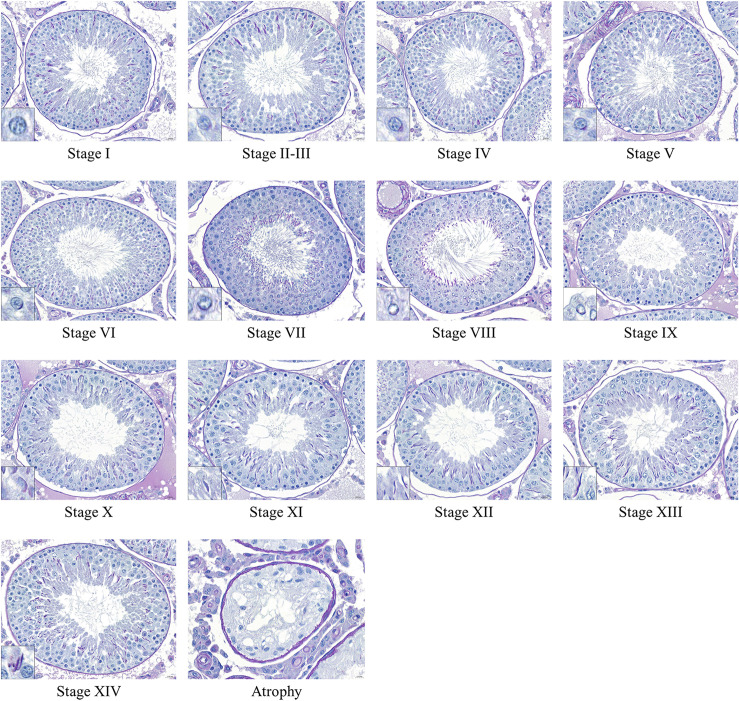
PAS-stained seminiferous tubules histology of the spermatogenic stage. The 14 stages were classified based on the shape of the acrosome and the spermatid. Stages II and III were combined owing to their indistinguishable appearance under microscopic observation. Atrophy was characterized by the depletion of germ cells and a reduction in the size of seminiferous tubules. The bottom-left box is a magnified view of the acrosome and round spermatids.

A total of 14 classes were identified, with spermatogenic stages II and III combined because of the difficulty in microscopic differentiation. Atrophy was included as an additional class. Classification of the stages was based on Russell’s standards [15]. The criteria for spermatogenic stages are summarized in Table 1. Fig 2 shows the histology of the spermatogenic stage. All annotations were performed by a single experienced pathologist according to predefined histopathological criteria (Table 1). To ensure consistency, annotations were conducted in multiple review rounds, and ambiguous cases were re-evaluated to maintain internal consistency.

**Table 1:** Criteria of the spermatogenic stage [15].

Stage	Criteria
I	No acrosome is visible in spermatids.
II–III	Proacrosomal or acrosomal granules are visible; the acrosome is not flat enough to form a hemisphere at the nuclear surface.
IV	Acrosomal vesicles begin to form a hemisphere at the nuclear surface, extending until it covers approximately 40 degrees of the nuclear surface.
V	The acrosome extends from 40 degrees to a maximum of 95 degrees.
VI	The acrosome extends from over 95 degrees to a maximum of 120 degrees; the elongated spermatid remains within deep crypts of Sertoli cells.
VII	Elongated spermatids align themselves linearly toward the luminal side of the seminiferous tubule epithelium; the angle of the acrosome is greater than 120 degrees; the nucleus of the round spermatid does not contact the plasma membrane.
VIII	The nucleus of a round spermatid contacts the plasma membrane, but the shape of the nucleus remains round or slightly oval without distortion.
IX	The nucleus of the round spermatid becomes elongated or oval; no ventral angle is evident.
X	The ventral angle of the spermatid is visible, but the dorsal angle is not apparent.
XI	Spermatid elongates further, and the dorsal angle becomes visible; the head of the spermatid is gently curved dorsally, but a bent-rod shape is not formed.
XII	Bent-rod shape begins to form; chromatin is not densely stained.
XIII	The densely stained portion extends throughout the entire head of the spermatid; this stage ends before metaphase I of meiotic division of diplotene spermatocytes begins.
XIV	Primary meiosis anaphase or telophase, secondary spermatocytes or secondary meiosis appear at this stage.
Atrophy	Only Sertoli cells remain, while germ cells are reduced or depleted.

### Deep learning model training

#### Dataset preparation.

The annotated 683 tile images from the 10 WSIs of 10 rats were randomly divided into train (70%, 478 images), valid (20%, 137 images), and test (10%, 68 images) datasets. The dataset was partitioned at the tile level for model training, validation, and testing. Consequently, tiles derived from the same animal may have been included across different datasets. This approach was selected to increase the number of training samples. Each image was downscaled to 2048 × 2048 × 3. Data augmentation techniques, including flipping (horizontal and vertical), 90-degree rotation (clockwise and counter-clockwise), cropping (0% minimum zoom and 25% maximum zoom), rotation (between −15° and +15°), shear (±10° horizontal and ±10° vertical), exposure (between −10% and +10%), and saturation (between −25% and +25%) adjustment, increased the training dataset to 1,434 images. A tile image contains multiple class objects as shown in Fig 1. Table 2 lists the 15,649 objects used for each dataset.

**Table 2:** Number of objects used in the models.

Stage	Train	Valid	Test
I	2458	198	118
II–III	1100	121	56
IV	624	73	41
V	649	77	27
VI	588	61	30
VII	2760	275	135
VIII	886	84	41
IX	439	45	24
X	402	33	17
XI	476	52	20
XII	1119	116	68
XIII	990	101	45
XIV	707	53	41
Atrophy	417	60	22
**Total**	13615	1349	685
	15649		

#### Comparison of model architecture.

**Fig 3:**
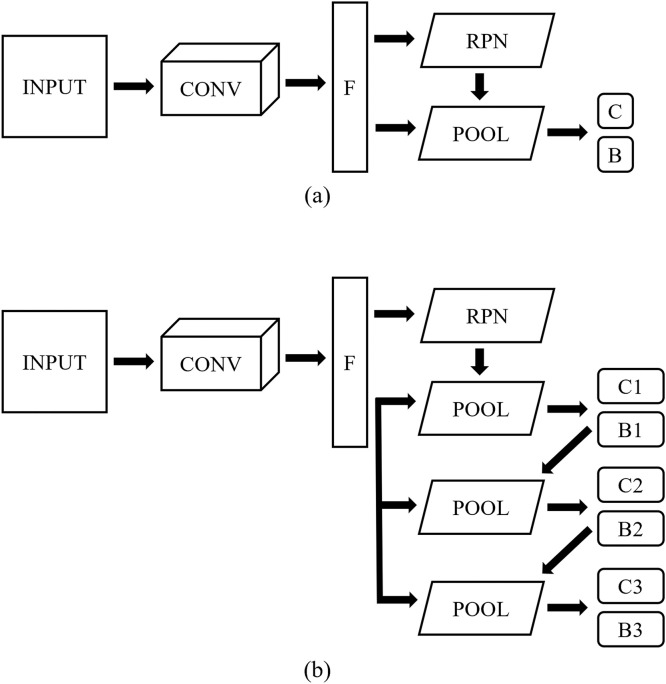
Faster R-CNN and Cascade R-CNN architectures. **(a)** Faster R-CNN architecture. The model directly and efficiently extracts region proposals from the feature map through RPN. **(b)** Cascade R-CNN architecture. The model extracts region proposals similar to Faster-RCNN, but it uses a three-stage detection process to increase precision. Abbreviations: CONV, convolutional layers; F, feature map; RPN, region proposal networks; POOL, RoI pooling; C, classification; B, bounding box regression.

Faster R-CNN replaces the inefficient Selective Search of Fast R-CNN with a Region Proposal Network that directly generates region proposals from the shared feature maps of the backbone CNN. The Region Proposal Network uses anchor boxes of various sizes and proportions to propose better regions efficiently. These proposals then go through Region of Interest Pooling and fully connected layers for the final classification and bounding box regression. Building on Faster R-CNN, Cascade R-CNN uses a multi-stage detection process to refine region proposal progressively. The first stage works as a typical Faster R-CNN detector, generating initial bounding box predictions. The second and third stages were trained using previously predicted boxes and a higher Intersection over Union (IoU) threshold than in the previous stages to reduce false positives. The simplified architectures of Faster R-CNN and Cascade R-CNN are shown in Fig 3.

#### Configuration settings.

The scale of the training and testing images was set to 2048 × 2048. To manage memory, the batch size was set to two for training and one for validation and testing. The number of epochs was set to 12 or 24, with validation performed after each epoch. The learning rate was set to 0.02/10, with a linear learning rate of 0.001 applied for the first 500 iterations. Additionally, a learning rate decay was applied twice during training. Further details regarding the settings are provided in S1 File. Model training was conducted using an RTX A4500 graphics processing unit (NVIDIA, Santa Clara, CA, USA) and the MMDetection toolbox [16].

### Model evaluation

#### Performance metrics.

Precision and recall metrics were evaluated using the test dataset. Precision represents the proportion of true positives among the sum of true positives and false positives, where recall (sensitivity) quantifies true positives among the sum of true positives and false negatives.


$$\begin{array}{c} \begin{array}{c} Precision=\frac{TP}{TP+FP}\;Recall=\frac{TP}{TP+FN} \end{array} \end{array}$$ (1)

The Precision-Recall (PR) curve illustrates the precision and recall variations according to confidence score thresholds. The Average Precision (AP), which is the area under the PR curve, evaluates the model’s performance across confidence score thresholds. In multi-class scenarios, the mean Average Precision (mAP) is calculated. Similarly, mean Average Recall (mAR) represents the mean recall across IoU thresholds.

#### Whole-slide image inference.

To verify practicality, inference was conducted on six WSIs not used in training or performance evaluation, including three normal (named normal A, B, and C) and three atrophied testes (severity of minimal, mild, and severe). Atrophy severity was defined as minimal (<10%), mild (10–30%), moderate (30–50%), or severe (>50%). Owing to the large WSI size, the SAHI library [17] was employed for memory-efficient inference. The inference was conducted using level 2 slide images, which provided the second-highest resolution among the 18 available levels. Owing to computational memory constraints, level 2 was selected as the optimal balance between the resolution and processing capability. After WSI inference, the results of normal WSIs were compared with the evaluation of pathologist 1, who provided the ground truth.

#### Stage frequency and statistics.

Stage frequencies were analyzed across the model, three pathologists (named pathologists 1, 2, and 3), and published literature by Hess et al. [18]. Pathologist 1 was the ground truth provider who performed image annotation, and two pathologists, 2 and 3, were independent pathologists unrelated to the ground truth. Statistical analyses were performed using IBM SPSS Statistics. For each sample, the proportion of seminiferous tubules assigned to each spermatogenic stage was calculated, and these sample-level proportions were used for group-level comparisons. Multivariate analysis of variance was applied in an exploratory manner to assess overall differences in stage-wise distributions between groups, assuming approximate multivariate normality and homogeneity of covariance matrices. When a significant multivariate effect was observed, Dunnett’s post hoc test was used to perform multiple comparisons while controlling for type I error, with the model results serving as the reference group. A two-sided p-value < 0.05 was considered statistically significant.

## Results

### Model performance metrics

The performance of the models was evaluated using the test dataset. Comparisons were made between two model types, Faster R-CNN and Cascade R-CNN, with two backbone options (ResNet-50 and ResNet-101) and two training durations (12 and 24 epochs). As shown in Table 3, the best-performing model was the Cascade R-CNN with a ResNet-50 backbone trained for 12 epochs. Increasing the backbone depth or extending the training duration did not significantly improve performance. When the IoU threshold range was set to 0.50:0.95, the best model achieved anmAP of 0.869 and an mAR of 0.977.

**Table 3:** Performance results of the models.

Model type	Backbone	Epochs	mAP^a^	mAR^a^
Faster R-CNN	ResNet-50	12	0.824	0.947
Faster R-CNN	ResNet-101	12	0.807	0.959
Faster R-CNN	ResNet-101	24	0.836	0.963
Cascade R-CNN	ResNet-50	12	0.869	0.977
Cascade R-CNN	ResNet-101	12	0.867	0.977
Cascade R-CNN	ResNet-101	24	0.857	0.960

Abbreviations: mAP, mean Average Precision; mAR, mean Average Recall.

^a^IoU = 0.50:0.95.

**Fig 4:**
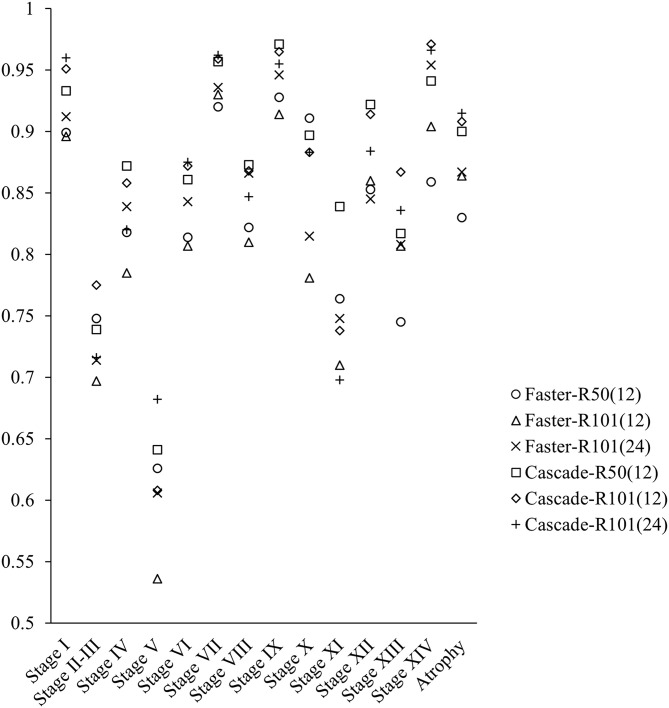
A dot plot showing stage-wise AP results of six models. The results of the Cascade R-CNN were generally higher than those of the Faster R-CNN. Legend: R is an abbreviation for ResNet, and the numbers in parentheses represent epochs.

The result of stage-wise AP is represented in Fig 4 and S1 Table. The AP of stages II–III, V, and XI was relatively lower. In case of atrophy detection, the AP tended to be high, exceeding 0.9, especially when using the Cascade R-CNN models.

### Whole slide image inference for spermatogenic staging

The best-performing model (Cascade R-CNN with a ResNet-50 backbone) was used for WSI inference. The average inference time for three normal testicular WSIs was 211 seconds. Detailed inference time is presented in Table 4.

**Table 4:** WSI inference times for three normal testicular WSIs.

WSI	Time (seconds)
Normal A	216.01
Normal B	231.38
Normal C	185.80
**Average**	211.06

**Fig 5:**
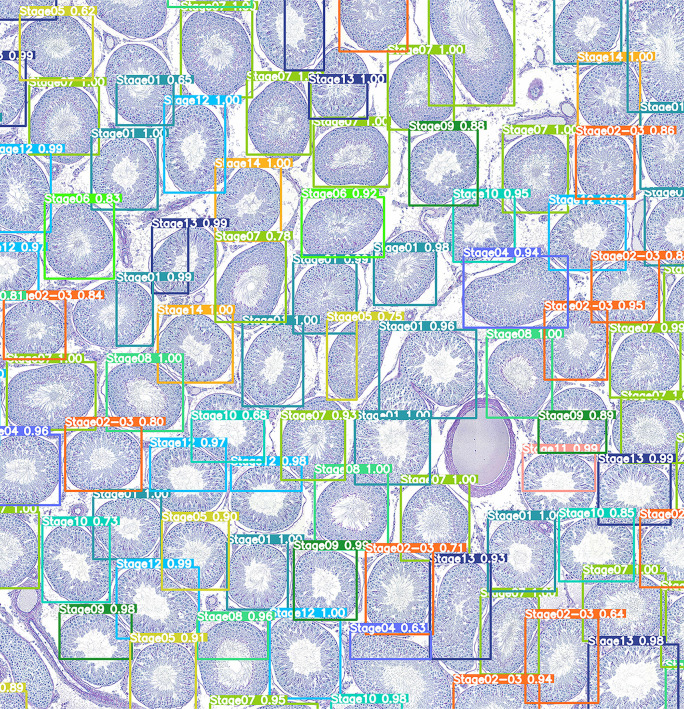
Partial inference result image from the Normal A WSI. Individual seminiferous tubules were identified and classified by stage using color-coded bounding boxes.

**Fig 6:**
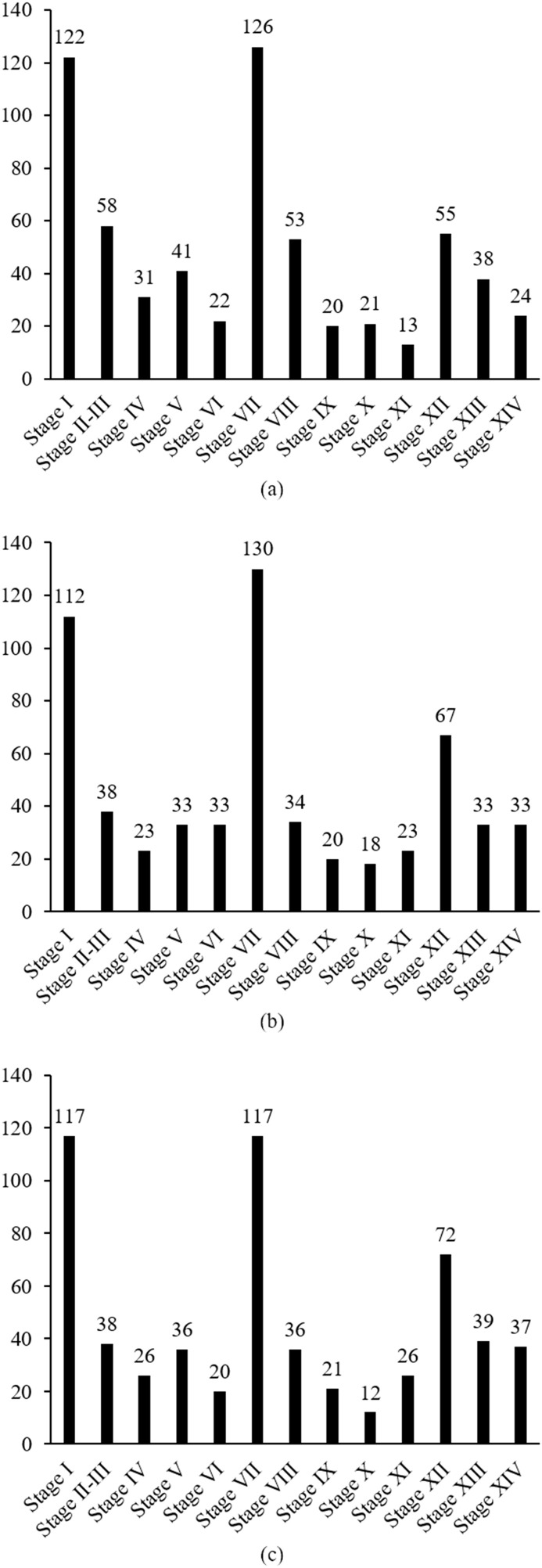
Bar graphs showing the inference results from the Normal A, B, and C WSIs. The graphs represent the number of inferred stages (i.e., the number of bounding boxes) in each WSI. **(a)** Inference result from the Normal A WSI. **(b)** Inference result from the Normal B WSI. **(c)** Inference result from the Normal C WSI.

As shown in Figs 5 and 6, the model successfully detected seminiferous tubules in normal testis WSIs. However, it occasionally produced duplicate inferences for longitudinally sectioned tubules. Duplicate bounding boxes accounted for an average of 1.92% of detections, whereas undetected tubules accounted for an average of 1.37%. Detailed proportion and count of duplicate and undetected boxes are listed in Table 5.

**Table 5:** Proportion and count of duplicate bounding boxes and undetected tubules in three normal testicular WSI inference results.

WSI (total count)	Duplicate (count)	Undetected (count)
Normal A (624)	2.40% (15)	1.44% (9)
Normal B (597)	1.34% (8)	1.50% (9)
Normal C (597)	2.01% (12)	1.17% (7)
**Average**	1.92%	1.37%

**Fig 7:**
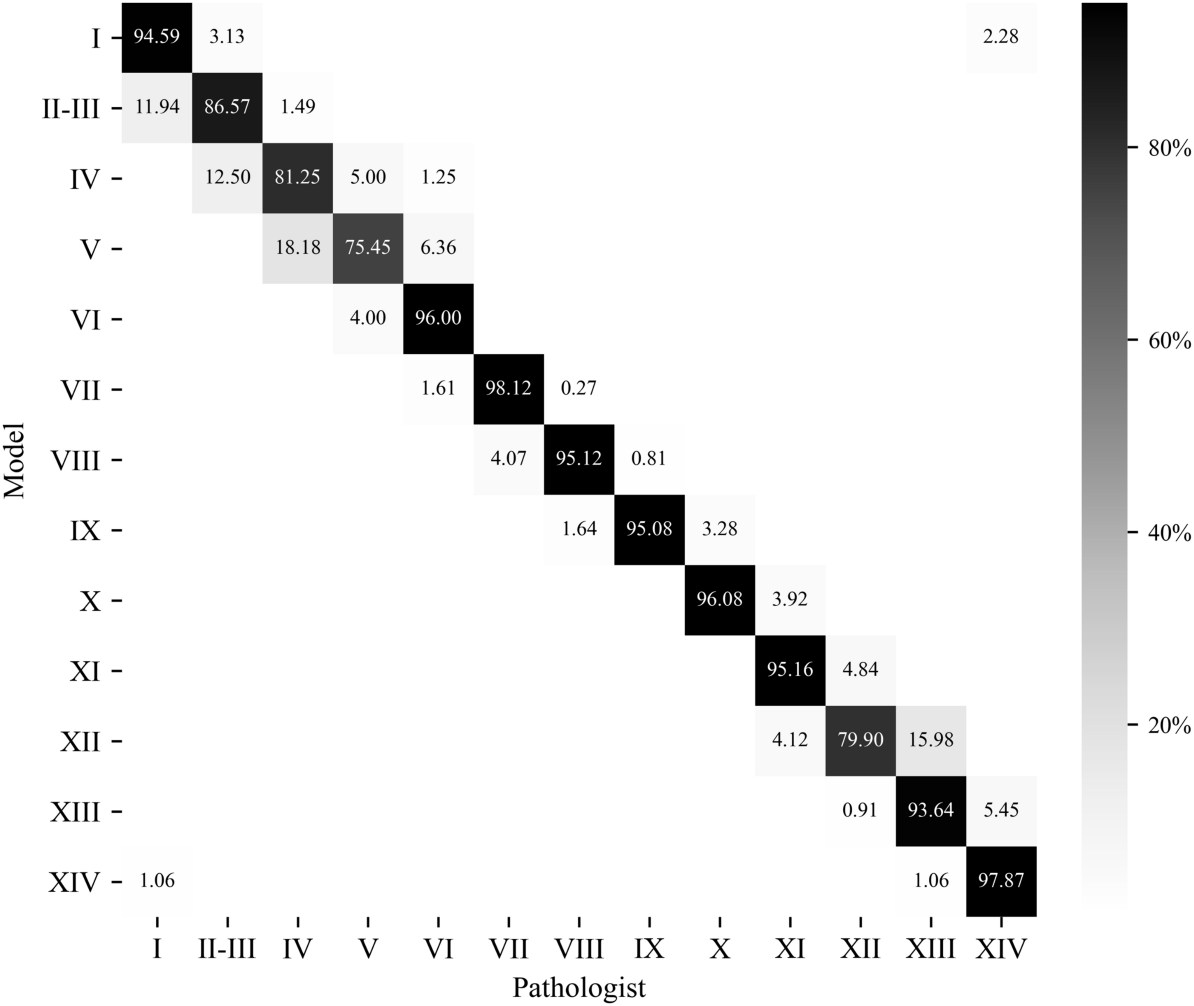
Confusion matrix comparing results of the model and the pathologist 1 in three normal testicular WSIs. The percentages represent precision (the proportion of true positive among the sum of true positive and false positive). Most stages demonstrated high accuracy, exceeding 90%, and no significant deviations from the ground truth were observed in the model’s output.

Additionally, the model results were compared with pathologist 1’s assessments, which provided the ground truth, using a confusion matrix, as shown in Fig 7. Detailed values for the three slides are presented in S2 Table. Atrophied tubules near the rete testis were considered normal. Therefore cases of atrophy detected in normal testes were excluded from the bar graph and confusion matrix results. The mean confusion matrix accuracy was 91%. Agreement rates of 92.0%, 90.0%, and 93.1% were observed across three independently inferred WSIs. These results demonstrate comparable performance across individual WSIs. Mean confusion matrix accuracy was higher than the mAP values due to the differences in evaluation criteria. Unlike AP calculations that rely on IoU and confidence scores, the confusion matrix considers a classification as correct if the assigned label is accurate, even when the bounding box overlap is partial.

**Fig 8:**
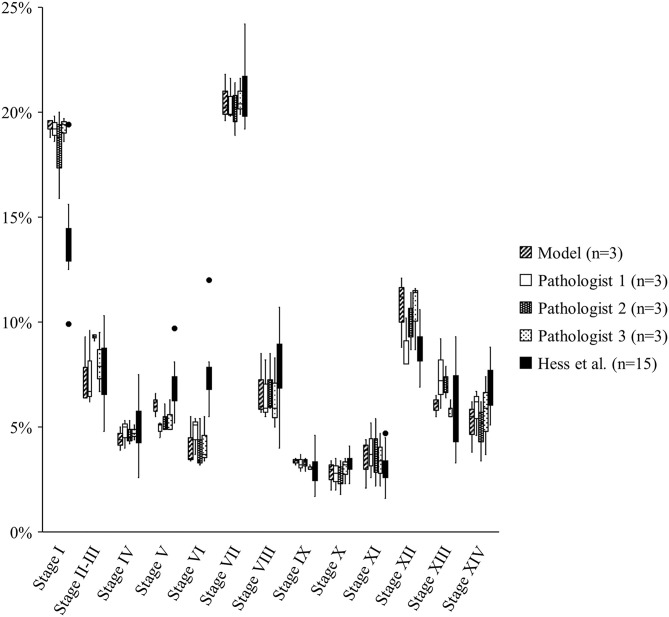
A box-and-whisker plot showing stage frequencies of the model, three pathologists, and the literature. The model and pathologists 1, 2, and 3 showed similar results, whereas differences were observed between the model and Hess et al.‘s results in stages I, VI, and XII. This study evaluated spermatogenic stages in three testes, while the research by Hess et al. (1990) assessed stages in 15 testes. Pathologist 1 served as the ground truth, while pathologists, 2 and 3, were independent pathology experts not associated with the ground truth.

A comparison of the stage frequencies across the model, the three pathologists, and the literature by Hess et al. is depicted in Fig 8. Detailed values of the stage frequencies are listed in S3 Table. When comparing the mean differences using statistical analysis, significant differences were found in the frequencies of stages I, VI, and XII between the model and literature. However, no significant differences were observed between the model and pathologists. Detailed values of statistics are presented in S4 Table.

### Whole slide image inference for atrophy detection

**Fig 9:**
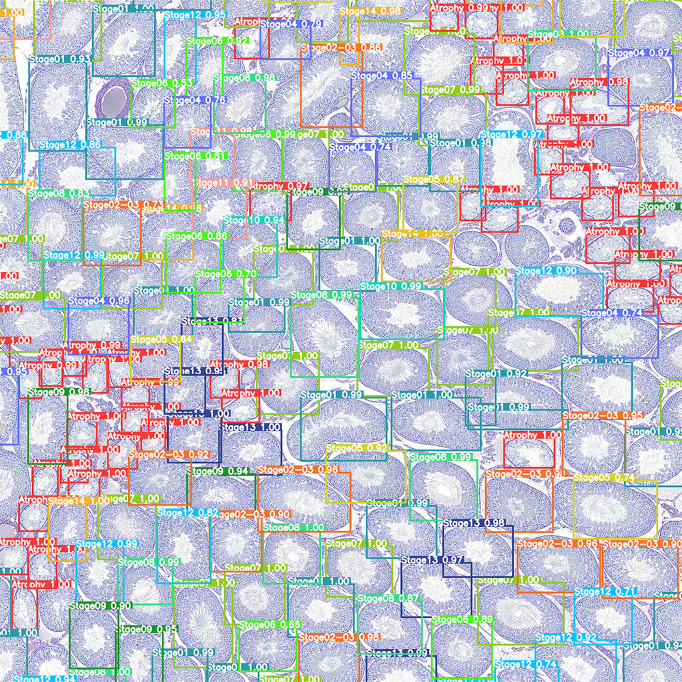
Partial inference result image from the minimally atrophied testicular WSI. Atrophied seminiferous tubules are indicated by red bounding boxes.

**Fig 10:**
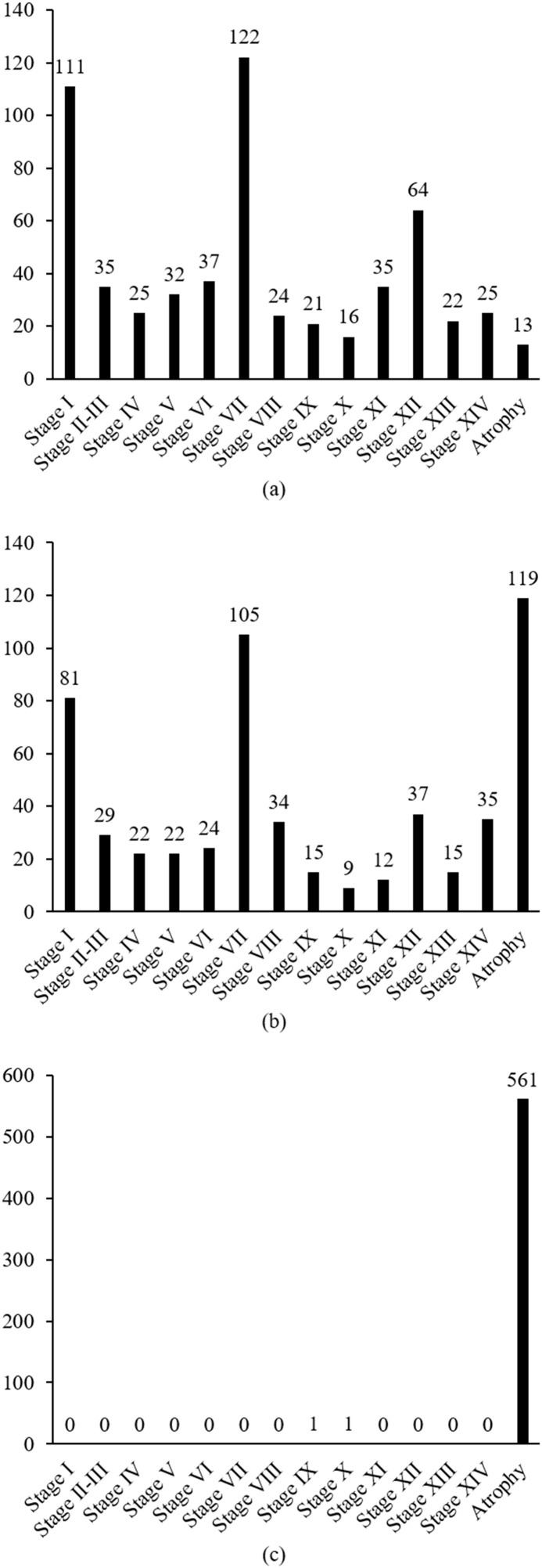
Bar graphs showing inference results from WSIs with three degrees of atrophy. The degree of atrophy can be quantitatively classified into minimal (<10%), mild (10–30%), and severe (>50%) based on the number of atrophied structures. **(a)** Inference result from the minimally atrophied WSI. **(b)** Inference results from the mildly atrophied WSI. **(c)** Inference results from the severely atrophied WSI.

For quantitative analysis of atrophy, inference was performed on three WSI cases with different degrees of atrophy. As shown in Figs 9 and 10, the degree of atrophy could be quantitatively assessed by counting the number of atrophied seminiferous tubules identified by the model. In a severely atrophied testicular WSI, where all seminiferous tubules exhibited atrophy, only two misclassifications were observed.

## Discussion

This study evaluated the feasibility and biological validity of an object detection–based deep learning framework for spermatogenic stage classification in rat testicular histopathology. By directly modeling seminiferous tubules and assigning all spermatogenic stages at the tubule level, the proposed approach was designed to overcome key limitations of prior segmentation-based or binary classification methods, particularly in the context of large-scale nonclinical reproductive toxicity studies.

The overall performance profile, characterized by a relatively high mAR combined with a lower stage-wise AP for the selected stages, suggests that the model favors sensitivity over specificity. This indicates an increased number of false-positive detections rather than a systematic failure to identify seminiferous tubules. In this object detection–based staging framework, most false positives correspond to correctly localized seminiferous tubules with incorrect stage assignments or duplicate detections, rather than spurious detection of background regions. Such a performance trade-off is expected in histopathological contexts where morphological boundaries between classes are inherently continuous rather than discrete. Importantly, this recall-precision trade-off was not uniformly distributed across stages but showed a stage-dependent pattern, prompting closer examination of the underlying biological determinants.

In this context, the lower precision observed for stages II–III, V, and XI can be largely explained by biological and anatomical factors rather than by technical instability. Stages II–III represent an early transitional period marked by the initial formation of the acrosome, which may be visible only in limited regions of the tubule and is highly sensitive to sectioning orientation. Stage V exhibited the lowest precision, which is consistent with its wide acrosomal angle range and substantial morphological overlap with adjacent stages IV and VI. Importantly, accurate evaluation of acrosomal angles is challenging even for experienced pathologists, as assessments are often based on partial or obliquely sectioned views. Misclassification involving stage XI likely reflects its short duration within the spermatogenic cycle and the frequent coexistence of spermatids at different maturation states within individual tubules. Collectively, these error patterns align with known biological continua in spermatogenesis and mirror the challenges inherent in manual staging.

Beyond technical performance, the distinction between multi-class and binary classifications has important implications for toxicological interpretations. Although binary classification schemes (e.g., normal versus atrophic tubules) are simpler and often sufficient for detecting overt toxicity, they inherently obscure the cyclic and stage-specific nature of spermatogenesis. Spermatogenic disruption can manifest as selective vulnerability at specific developmental stages, such as endocrine-mediated arrest at defined transitions, rather than uniform degeneration across the entire tubule population. For example, testosterone has been reported to influence the transition from stage VII to VIII of the seminiferous epithelium cycle [19,20]. Multi-class staging enables the localization of toxic effects along the spermatogenic continuum and supports mechanistic interpretation by distinguishing between pre-meiotic, meiotic, and post-meiotic alterations. In this context, the ability to resolve individual stages provides substantially greater biological and toxicological insight than binary categorization.

Although multi-class classification of spermatogenic stages is inherently challenging owing to high inter-stage similarity, previous studies have demonstrated its feasibility using segmentation-heavy pipelines combined with rule-based or decision tree classifiers. However, these approaches are computationally intensive and often collapse stages into coarse categories when applied to H&E-stained sections. The present object detection–based strategy achieves full stage resolution while reducing computational complexity, thereby enhancing practical applicability without sacrificing biological granularity.

However, accurate multi-class staging critically depends on the visibility of acrosomal features, underscoring the importance of staining methodology. PAS staining is a critical methodological choice. In rat testes, the spermatogenic stages are primarily defined by the development, orientation, and morphology of the spermatid acrosome, a glycoprotein-rich structure that is optimally visualized by PAS staining. In contrast, H&E staining provides limited contrast for acrosomal features, particularly in transitional stages, thereby constraining reliable stage discrimination. By enhancing visualization of acrosomal formation and migration, PAS staining improves both manual annotation accuracy and model-based classification fidelity.

Whole-slide inference required approximately 211 seconds per slide, corresponding to less than 1.5 hours for a typical multi-animal reproductive toxicity study. This level of efficiency supports the feasibility of routine or large-scale toxicological applications, particularly when compared with manual staging, which is labor-intensive and often limited to a small subset of tubules. An exhaustive whole-slide analysis enables more representative assessment of stage distributions and reduces sampling bias inherent to partial manual evaluation.

Stage-resolved frequency analysis revealed deviations from previously reported distributions for specific stages, including I, VI, and XII. These discrepancies did not present as reciprocal shifts between morphologically adjacent stages, suggesting that they are unlikely to arise from systematic misclassification. Instead, they may reflect biological variability, such as age or inter-individual differences, or technical variability, such as staining procedures or annotation subjectivity, compounded by the limited number of animals analyzed in this study and in the reference literature. For regulatory and screening purposes, reproducible detection of relative, stage-specific shifts between the treated and control groups is often of greater relevance than exact concordance with historical absolute frequencies.

The framework demonstrated a robust performance in identifying and quantifying atrophied seminiferous tubules, supporting objective severity assessments of pathological conditions. Conventional grading of testicular atrophy often relies on subjective estimation of affected areas, whereas quantitative tubule-level analysis provides a reproducible alternative. By enabling observer-independent, stage-resolved analysis at scale, the proposed approach addresses key limitations of current nonclinical reproductive toxicity assessments, including labor intensity, subjectivity, and limited standardization across studies.

A key concern relates to potential overfitting and generalizability, particularly given that the training data included images derived from a limited number of animals and a single atrophied testis. Additionally, because data partitioning was performed at the tile level, images originating from the same animal could be included across different datasets, which may further limit strict animal-level independence. Several design choices partially mitigate this risk. Training was conducted at the object (tubule) level, yielding a large number of independent instances per slide and capturing substantial intra-slide morphological variability. Extensive data augmentation was applied to increase appearance diversity and reduce reliance on slide-specific features. Additionally, class imbalance was implicitly addressed through proposal-level sampling and IoU-based supervision inherent to two-stage detectors, as well as multi-stage refinement in the Cascade R-CNN architecture. Nonetheless, animal-level independence cannot be fully guaranteed, and the inclusion of a single atrophied testis limits conclusions regarding lesion variability. These limitations underscore the need for future validation using larger and more diverse cohorts, multiple pathological specimens, and animal-wise data partitioning to assess generalizability more rigorously.

## Conclusion

This study demonstrates that an object detection–based deep learning framework enables efficient and biologically meaningful spermatogenic stage classification in PAS-stained rat testicular WSIs. From a research perspective, the proposed approach allows stage-resolved frequency analysis across all testes, facilitating quantitative comparison of spermatogenic patterns and detection of subtle, stage-specific perturbations relevant to mechanistic toxicology. From a practical standpoint, automated tubule-level classification and atrophy quantification reduce manual workload and improve consistency in routine histopathological evaluations. Although further validation in larger and more diverse cohorts is required, the framework provides a scalable foundation for high-throughput reproductive toxicity studies and may be extended to other testicular pathologies or integrated into decision-support tools for preclinical and translational research.

## Supporting information

S1 Table Stage-wise AP result of six models. Abbreviations: AP, Average Precision, AR, Average Recall.
(PDF)

S2 Table Confusion matrix between the model and pathologist 1 (ground truth). (a) Confusion matrix of normal A testicular WSI. (b) Confusion matrix of normal B testicular WSI. (c) Confusion matrix of normal C testicular WSI. (d) Confusion matrix of the sum of all three normal testicular WSIs.
(PDF)

S3 Table Stage frequency values for the evaluation results of the model, three pathologists, and Hess et al. Spermatogenic stage frequencies were calculated from normal testicular tissues. (PDF)

S4 Table Statistics of stage frequency. Significance was assessed when the p-value of the mean difference was less than 0.05.
(PDF)

S1 File System environment and configuration settings. (PDF)
