# Supplementary material for: Automated spermatogenic staging in periodic acid-Schiff-stained testes of Sprague–Dawley rats using a deep learning model for normal and atrophied tissues
Source: PLoS One. 2026 Jun 29;21(6):e0337245. doi: 10.1371/journal.pone.0337245 (PMC13313349; doi:10.1371/journal.pone.0337245)
Supplement: S1 Table — Abbreviations: AP, Average Precision, AR, Average Recall. (PDF) [file pone.0337245.s001.pdf]

Model type	Backbone	Epochs	AP	AR	I	II-III	IV	V	VI	VII	VIII	IX	X	XI	XII	XIII	XIV	Atrophy
Faster R-CNN	Resnet50	12	0.824	0.947	0.899	0.748	0.818	0.626	0.814	0.92	0.822	0.928	0.911	0.764	0.853	0.745	0.859	0.83
Faster R-CNN	Resnet101	12	0.807	0.959	0.896	0.697	0.785	0.536	0.807	0.93	0.81	0.914	0.781	0.71	0.86	0.807	0.904	0.864
Faster R-CNN	Resnet101	24	0.836	0.963	0.912	0.714	0.839	0.606	0.843	0.936	0.866	0.946	0.815	0.748	0.845	0.808	0.954	0.867
Cascade R-CNN	Resnet50	12	0.869	0.977	0.933	0.739	0.872	0.641	0.861	0.957	0.873	0.971	0.897	0.839	0.922	0.817	0.941	0.9
Cascade R-CNN	Resnet101	12	0.867	0.977	0.951	0.775	0.858	0.608	0.872	0.959	0.868	0.965	0.883	0.738	0.914	0.867	0.971	0.908
Cascade R-CNN	Resnet101	24	0.857	0.96	0.96	0.716	0.82	0.682	0.875	0.962	0.847	0.955	0.883	0.698	0.884	0.836	0.966	0.915
