# Supplementary material for: Automated spermatogenic staging in periodic acid-Schiff-stained testes of Sprague–Dawley rats using a deep learning model for normal and atrophied tissues
Source: PLoS One. 2026 Jun 29;21(6):e0337245. doi: 10.1371/journal.pone.0337245 (PMC13313349; doi:10.1371/journal.pone.0337245)
Supplement: S2 Table — (a) Confusion matrix of normal A testicular WSI. (b) Confusion matrix of normal B testicular WSI. (c) Confusion matrix of normal C testicular WSI. (d) Confusion matrix of the sum of all three normal testicular WSIs. (PDF) [file pone.0337245.s002.pdf]

(a)

**Normal A**	Pathologist 1														
	Stage	I	II-III	IV	V	VI	VII	VIII	IX	X	XI	XII	XIII	XIV	A
Model	I	114	6	0	0	0	0	0	0	0	0	0	0	2	0
	III	6	51	1	0	0	0	0	0	0	0	0	0	0	0
	IV	0	3	27	0	1	0	0	0	0	0	0	0	0	0
	V	0	0	5	31	5	0	0	0	0	0	0	0	0	0
	VI	0	0	0	1	21	0	0	0	0	0	0	0	0	0
	VII	0	0	0	0	5	121	0	0	0	0	0	0	0	0
	VIII	0	0	0	0	0	3	50	0	0	0	0	0	0	0
	IX	0	0	0	0	0	0	1	18	1	0	0	0	0	0
	X	0	0	0	0	0	0	0	0	21	0	0	0	0	0
	XI	0	0	0	0	0	0	0	0	0	13	0	0	0	0
	XII	0	0	0	0	0	0	0	0	0	3	49	3	0	0
	XIII	0	0	0	0	0	0	0	0	0	0	1	34	3	0
	XIV	0	0	0	0	0	0	0	0	0	0	0	0	24	0
	A	0	0	0	0	0	0	0	0	0	0	0	0	0	3

(b)

**Normal B**	Pathologist 1														
	Stage	I	II-III	IV	V	VI	VII	VIII	IX	X	XI	XII	XIII	XIV	A
Model	I	104	4	0	0	0	0	0	0	0	0	0	0	4	0
	III	7	31	0	0	0	0	0	0	0	0	0	0	0	0
	IV	0	2	18	3	0	0	0	0	0	0	0	0	0	0
	V	0	0	6	26	1	0	0	0	0	0	0	0	0	0
	VI	0	0	0	2	31	0	0	0	0	0	0	0	0	0
	VII	0	0	0	0	0	129	1	0	0	0	0	0	0	0
	VIII	0	0	0	0	0	0	34	0	0	0	0	0	0	0
	IX	0	0	0	0	0	0	0	19	1	0	0	0	0	0
	X	0	0	0	0	0	0	0	0	16	2	0	0	0	0
	XI	0	0	0	0	0	0	0	0	0	20	3	0	0	0
	XII	0	0	0	0	0	0	0	0	0	0	45	22	0	0
	XIII	0	0	0	0	0	0	0	0	0	0	0	32	1	0
	XIV	0	0	0	0	0	0	0	0	0	0	0	1	32	0
	A	0	0	0	0	0	0	0	0	0	0	0	0	0	2

(c)

Normal C	Pathologist 1														
	Stage	I	II-III	IV	V	VI	VII	VIII	IX	X	XI	XII	XIII	XIV	A
	I	114	1	0	0	0	0	0	0	0	0	0	0	2	0
	III	3	34	1	0	0	0	0	0	0	0	0	0	0	0
	IV	0	5	20	1	0	0	0	0	0	0	0	0	0	0
	V	0	0	9	26	1	0	0	0	0	0	0	0	0	0
	VI	0	0	0	0	20	0	0	0	0	0	0	0	0	0
	VII	0	0	0	0	1	116	0	0	0	0	0	0	0	0
Model	VIII	0	0	0	0	0	2	33	1	0	0	0	0	0	0
	IX	0	0	0	0	0	0	0	21	0	0	0	0	0	0
	X	0	0	0	0	0	0	0	0	12	0	0	0	0	0
	XI	0	0	0	0	0	0	0	0	0	26	0	0	0	0
	XII	0	0	0	0	0	0	0	0	0	5	61	6	0	0
	XIII	0	0	0	0	0	0	0	0	0	0	0	37	2	0
	XIV	1	0	0	0	0	0	0	0	0	0	0	0	36	0
	A	0	0	0	0	0	0	0	0	0	0	0	0	0	1

(d)

Sum	Pathologist 1														
	Stage	I	II-III	IV	V	VI	VII	VIII	IX	X	XI	XII	XIII	XIV	A
	I	332	11	0	0	0	0	0	0	0	0	0	0	8	0
	III	16	116	2	0	0	0	0	0	0	0	0	0	0	0
	IV	0	10	65	4	1	0	0	0	0	0	0	0	0	0
	V	0	0	20	83	7	0	0	0	0	0	0	0	0	0
	VI	0	0	0	3	72	0	0	0	0	0	0	0	0	0
	VII	0	0	0	0	6	366	1	0	0	0	0	0	0	0
Model	VIII	0	0	0	0	0	5	117	1	0	0	0	0	0	0
	IX	0	0	0	0	0	0	1	58	2	0	0	0	0	0
	X	0	0	0	0	0	0	0	0	49	2	0	0	0	0
	XI	0	0	0	0	0	0	0	0	0	59	3	0	0	0
	XII	0	0	0	0	0	0	0	0	0	8	155	31	0	0
	XIII	0	0	0	0	0	0	0	0	0	0	1	103	6	0
	XIV	1	0	0	0	0	0	0	0	0	0	0	1	92	0
	A	0	0	0	0	0	0	0	0	0	0	0	0	0	6
