# Supplementary material for: Automated spermatogenic staging in periodic acid-Schiff-stained testes of Sprague–Dawley rats using a deep learning model for normal and atrophied tissues
Source: PLoS One. 2026 Jun 29;21(6):e0337245. doi: 10.1371/journal.pone.0337245 (PMC13313349; doi:10.1371/journal.pone.0337245)
Supplement: S3 Table — (PDF) [file pone.0337245.s003.pdf]

Method	Slide	Stage I	Stage II-III	Stage IV	Stage V	Stage VI	Stage VII	Stage VIII	Stage IX	Stage X	Stage XI	Stage XII	Stage XIII	Stage XIV
Model	Normal A	19.6	9.3	5	6.6	3.5	20.2	8.5	3.2	3.4	2.1	8.8	6.1	3.8
Model	Normal B	18.8	6.4	3.9	5.5	5.5	21.8	5.7	3.4	3	3.9	11.2	5.5	5.5
Model	Normal C	19.6	6.4	4.4	6	3.4	19.6	6	3.5	2	4.4	12.1	6.5	6.2
P1	Normal A	19.2	9.6	5.3	5.1	5.1	19.9	8.2	2.9	3.5	2.6	8.0	5.9	4.6
P1	Normal B	18.6	6.2	4.0	5.2	5.4	21.6	5.9	3.2	2.8	3.7	8.0	9.2	6.2
P1	Normal C	19.8	6.7	5.0	4.5	3.7	19.8	5.5	3.7	2.0	5.2	10.2	7.2	6.7
P2	Normal A	20.0	9.1	5.3	6.1	3.4	20.2	8.5	2.9	3.4	2.2	8.7	6.9	3.4
P2	Normal B	15.9	9.4	4.5	4.9	5.4	21.4	5.9	3.4	2.8	3.5	9.9	7.9	5.2
P2	Normal C	18.8	9.4	4.2	4.9	3.2	18.9	6.0	3.5	1.8	5.4	11.4	6.4	6.2
P3	Normal A	19.7	9.5	5.1	6.3	3.4	20.4	8.3	3	3.5	2.2	8.7	6.3	3.7
P3	Normal B	18.6	6.7	4.4	4.9	5.5	21.6	5.9	3.2	3.2	3.4	11.4	5.5	5.9
P3	Normal C	19.4	7.9	4.7	4.9	3.7	19.9	5	3	2.3	4.7	11.6	5.5	7.4
Hess et al.	-	19.4	4.8	2.6	6.4	7.3	24.2	6.1	3.1	2.9	2.8	8.4	4.5	7.6
Hess et al.	-	14.6	6.2	3	7.7	6.6	22.5	9.6	2.8	3.6	3.6	7.3	6.4	6.4
Hess et al.	-	12.9	7.7	6.2	7.1	6.7	19.9	10.7	2.3	3.2	4.5	9.3	4.1	5.4
Hess et al.	-	13.1	6.6	4.3	8.1	8.1	21.8	7.4	3.8	3.1	3	8.1	5.5	7.1
Hess et al.	-	15	7.4	4.9	5.3	7.1	20.9	9.1	2.9	3.2	3	8.1	7.2	6.1
Hess et al.	-	13.8	9.9	5.8	6.2	7.2	21.2	6	2.1	2.9	4.7	9.4	3.3	7.2
Hess et al.	-	13.3	7.4	5.6	6.5	7.8	19.5	7.1	3.4	3.6	3.6	9.3	7.7	5.1
Hess et al.	-	14.3	6.9	4.8	6.6	5.9	20.8	6.6	4.6	4	2.6	8.2	6.7	8
Hess et al.	-	12.9	5.4	4.7	6.5	6.9	21	10	2.1	4.1	2.6	9	8.6	6.2
Hess et al.	-	12.5	8.9	5.7	6.5	7.9	19.3	8.8	3.9	3.4	3	8.8	6.2	5.2
Hess et al.	-	14	6.5	4.4	5.2	7.6	19.7	7.8	3.3	2.9	3.1	9	8.8	7.8
Hess et al.	-	9.9	10.3	6.4	8	7.5	21.9	7.1	1.7	3.1	1.6	10.6	4	8
Hess et al.	-	9.9	8.6	7.5	9.7	5.5	21.6	7.1	2.6	3.1	2.6	9.4	3.6	8.8
Hess et al.	-	15.6	8	4.2	6.3	8	20.3	7.3	2.7	2.3	1.8	6.9	9.3	7.3
Hess et al.	-	14.1	9.8	4	6.1	12	19.2	4	2.7	3.2	3.2	8.6	6.5	6
