# Supplementary material for: Automated spermatogenic staging in periodic acid-Schiff-stained testes of Sprague–Dawley rats using a deep learning model for normal and atrophied tissues
Source: PLoS One. 2026 Jun 29;21(6):e0337245. doi: 10.1371/journal.pone.0337245 (PMC13313349; doi:10.1371/journal.pone.0337245)
Supplement: S4 Table — Significance was assessed when the p-value of the mean difference was less than 0.05. (PDF) [file pone.0337245.s004.pdf]

Stage	Method	Mean	SEM	Mean difference	p-value
Stage I	Model	19.3	0.3	-	-
	Pathologist 1	19.2	0.3	-0.1	1.0
	Pathologist 2	18.2	1.2	-1.1	0.9
	Pathologist 3	19.2	0.3	-0.1	1.0
	Hess et al.	13.7	0.6	-5.647***	0.0
Stage II-III	Model	7.4	1.0	-	-
	Pathologist 1	7.5	1.1	0.1	1.0
	Pathologist 2	9.3	0.1	1.9	0.4
	Pathologist 3	8.0	0.8	0.6	1.0
	Hess et al.	7.6	0.4	0.3	1.0
Stage IV	Model	4.4	0.3	-	-
	Pathologist 1	4.8	0.4	0.3	1.0
	Pathologist 2	4.7	0.3	0.2	1.0
	Pathologist 3	4.7	0.2	0.3	1.0
	Hess et al.	4.9	0.3	0.5	0.9
Stage V	Model	6.0	0.3	-	-
	Pathologist 1	4.9	0.2	-1.1	0.5
	Pathologist 2	5.3	0.4	-0.8	0.7
	Pathologist 3	5.3	0.5	-0.7	0.8
	Hess et al.	6.8	0.3	0.8	0.5
Stage VI	Model	4.1	0.7	-	-
	Pathologist 1	4.7	0.5	0.6	0.9
	Pathologist 2	4.0	0.7	-0.2	1.0
	Pathologist 3	4.2	0.7	0.1	1.0
	Hess et al.	7.5	0.4	3.340**	0.0
Stage VII	Model	20.5	0.7	-	-
	Pathologist 1	20.4	0.6	-0.1	1.0
	Pathologist 2	20.2	0.7	-0.3	1.0
	Pathologist 3	20.6	0.5	0.1	1.0
	Hess et al.	20.9	0.4	0.4	1.0
Stage VIII	Model	6.7	0.9	-	-
	Pathologist 1	6.5	0.8	-0.2	1.0
	Pathologist 2	6.8	0.9	0.1	1.0
	Pathologist 3	6.4	1.0	-0.3	1.0
	Hess et al.	7.6	0.5	0.9	0.8
Stage IX	Model	3.4	0.1	-	-
	Pathologist 1	3.3	0.2	-0.1	1.0
	Pathologist 2	3.3	0.2	-0.1	1.0
	Pathologist 3	3.1	0.1	-0.3	0.9
	Hess et al.	2.9	0.2	-0.4	0.6
	Model	2.8	0.4	-	-
	Pathologist 1	2.8	0.4	0.0	1.0

Stage X	Pathologist 2	2.7	0.4	-0.1	1.0
	Pathologist 3	3.0	0.4	0.2	1.0
	Hess et al.	3.2	0.1	0.4	0.5
Stage XI	Model	3.5	0.7	-	-
	Pathologist 1	3.8	0.8	0.3	1.0
	Pathologist 2	3.7	0.9	0.2	1.0
	Pathologist 3	3.4	0.7	0.0	1.0
	Hess et al.	3.0	0.2	-0.4	0.9
Stage XII	Model	10.7	1.0	-	-
	Pathologist 1	8.8	0.7	-1.9	0.2
	Pathologist 2	10.0	0.8	-0.7	0.8
	Pathologist 3	10.5	0.9	-0.2	1.0
	Hess et al.	8.7	0.2	-2.007*	0.0
Stage XIII	Model	6.0	0.3	-	-
	Pathologist 1	7.4	1.0	1.4	0.7
	Pathologist 2	7.0	0.4	1.0	0.9
	Pathologist 3	5.8	0.3	-0.3	1.0
	Hess et al.	6.2	0.5	0.1	1.0
Stage XIV	Model	5.2	0.7	-	-
	Pathologist 1	5.8	0.6	0.7	0.9
	Pathologist 2	4.9	0.8	-0.2	1.0
	Pathologist 3	5.6	1.1	0.5	1.0
	Hess et al.	6.8	0.3	1.6	0.1

$^{*}p<0.05$, $^{**}p<0.01$, $^{***}p<0.001$
