# Supplementary material for: Automated spermatogenic staging in periodic acid-Schiff-stained testes of Sprague–Dawley rats using a deep learning model for normal and atrophied tissues
Source: PLoS One. 2026 Jun 29;21(6):e0337245. doi: 10.1371/journal.pone.0337245 (PMC13313349; doi:10.1371/journal.pone.0337245)
Supplement: S1 File — (PDF) [file pone.0337245.s005.pdf]

```
DATE - mmengine - INFO -
------------------------------------------------------------
System environment:
    sys.platform: win32
    Python: 3.8.19 | packaged by conda-forge | (default, Mar 20 2024, 
12:38:07) [MSC v.1929 64 bit (AMD64)]
    CUDA available: True
    MUSA available: False
    numpy_random_seed: 16523597
    GPU 0,1: NVIDIA RTX A4500
    CUDA_HOME: C:\Program Files\NVIDIA GPU Computing Toolkit\CUDA\v12.1
    NVCC: Cuda compilation tools, release 12.1, V12.1.66
    MSVC: Microsoft (R) C/C++ ÃÖÀûÈ- ÄÄÆÄÀÏ·¯ ¹öÀü 19.41.34120(x64)
    GCC: n/a
    PyTorch: 2.1.0
    PyTorch compiling details: PyTorch built with:
  - C++ Version: 199711
  - MSVC 192930151
  - Intel(R) Math Kernel Library Version 2020.0.2 Product Build 20200624 
for Intel(R) 64 architecture applications
  - Intel(R) MKL-DNN v3.1.1 (Git Hash 
64f6bcbcbab628e96f33a62c3e975f8535a7bde4)
  - OpenMP 2019
  - LAPACK is enabled (usually provided by MKL)
  - CPU capability usage: AVX2
  - CUDA Runtime 12.1
  - NVCC architecture flags: -gencode;arch=compute_50,code=sm_50;-
gencode;arch=compute_60,code=sm_60;-gencode;arch=compute_61,code=sm_61;-
gencode;arch=compute_70,code=sm_70;-gencode;arch=compute_75,code=sm_75;-
gencode;arch=compute_80,code=sm_80;-gencode;arch=compute_86,code=sm_86;-
gencode;arch=compute_90,code=sm_90
  - CuDNN 8.8.1  (built against CUDA 12.0)
  - Magma 2.5.4
  - Build settings: BLAS_INFO=mkl, BUILD_TYPE=Release, CUDA_VERSION=12.1, 
CUDNN_VERSION=8.8.1, 
CXX_COMPILER=C:/cb/pytorch_1000000000000/work/tmp_bin/sccache-cl.exe, 
CXX_FLAGS=/DWIN32 /D_WINDOWS /GR /EHsc /bigobj /FS -DUSE_PTHREADPOOL -
DNDEBUG -DUSE_KINETO -DLIBKINETO_NOCUPTI -DLIBKINETO_NOROCTRACER -
DUSE_FBGEMM -DUSE_XNNPACK -DSYMBOLICATE_MOBILE_DEBUG_HANDLE /utf-8 
/wd4624 /wd4068 /wd4067 /wd4267 /wd4661 /wd4717 /wd4244 /wd4804 /wd4273, 
LAPACK_INFO=mkl, PERF_WITH_AVX=1, PERF_WITH_AVX2=1, PERF_WITH_AVX512=1, 
TORCH_DISABLE_GPU_ASSERTS=OFF, TORCH_VERSION=2.1.0, USE_CUDA=ON, 
USE_CUDNN=ON, USE_EXCEPTION_PTR=1, USE_GFLAGS=OFF, USE_GLOG=OFF, 
USE_MKL=ON, USE_MKLDNN=ON, USE_MPI=OFF, USE_NCCL=OFF, USE_NNPACK=OFF, 
USE_OPENMP=ON, USE_ROCM=OFF, 

    TorchVision: 0.16.0
    OpenCV: 4.10.0
    MMEngine: 0.10.4

Runtime environment:
    cudnn_benchmark: False
    mp_cfg: {'mp_start_method': 'fork', 'opencv_num_threads': 0}
    dist_cfg: {'backend': 'nccl'}
```

```
    seed: 16523597
    Distributed launcher: none
    Distributed training: False
    GPU number: 1
------------------------------------------------------------

DATE - mmengine - INFO - Config:
backend_args = None
data_root = 'data_PATH'
dataset_type = 'CocoDataset'
default_hooks = dict(
    checkpoint=dict(
        rule='greater', save_best='coco/bbox_mAP',
type='CheckpointHook'),
    logger=dict(interval=50, type='LoggerHook'),
    param_scheduler=dict(type='ParamSchedulerHook'),
    sampler_seed=dict(type='DistSamplerSeedHook'),
    timer=dict(type='IterTimerHook'),
    visualization=dict(type='DetVisualizationHook'))
default_scope = 'mmdet'
device = 'cuda'
env_cfg = dict(
    cudnn_benchmark=False,
    dist_cfg=dict(backend='nccl'),
    mp_cfg=dict(mp_start_method='fork', opencv_num_threads=0))
gpu_ids = range(0, 2)
launcher = 'none'
load_from = 'pth_file_PATH'
log_level = 'INFO'
log_processor = dict(by_epoch=True, type='LogProcessor', window_size=50)
metainfo = dict(
    classes=(
        'Atrophy',
        'Stage01',
        'Stage02-03',
        'Stage04',
        'Stage05',
        'Stage06',
        'Stage07',
        'Stage08',
        'Stage09',
        'Stage10',
        'Stage11',
        'Stage12',
        'Stage13',
        'Stage14',
    ))
model = dict(
    backbone=dict(
        depth=50,
        frozen_stages=1,
        init_cfg=dict(checkpoint='torchvision://resnet50',
type='Pretrained'),
        norm_cfg=dict(requires_grad=True, type='BN'),
```

```
        norm_eval=True,
        num_stages=4,
        out_indices=(
            0,
            1,
            2,
            3,
        ),
        style='pytorch',
        type='ResNet'),
    data_preprocessor=dict(
        bgr_to_rgb=True,
        mean=[
            123.675,
            116.28,
            103.53,
        ],
        pad_size_divisor=32,
        std=[
            58.395,
            57.12,
            57.375,
        ],
        type='DetDataPreprocessor'),
    neck=dict(
        in_channels=[
            256,
            512,
            1024,
            2048,
        ],
        num_outs=5,
        out_channels=256,
        type='FPN'),
    roi_head=dict(
        bbox_head=[
            dict(
                bbox_coder=dict(
                    target_means=[
                        0.0,
                        0.0,
                        0.0,
                        0.0,
                    ],
                    target_stds=[
                        0.1,
                        0.1,
                        0.2,
                        0.2,
                    ],
                    type='DeltaXYWHBBoxCoder'),
                fc_out_channels=1024,
                in_channels=256,
```

```
                loss_bbox=dict(beta=1.0, loss_weight=1.0,
type='SmoothL1Loss'),
                loss_cls=dict(
                    loss_weight=1.0,
                    type='CrossEntropyLoss',
                    use_sigmoid=False),
                num_classes=14,
                reg_class_agnostic=True,
                roi_feat_size=7,
                type='Shared2FCBBoxHead'),
            dict(
                bbox_coder=dict(
                    target_means=[
                        0.0,
                        0.0,
                        0.0,
                        0.0,
                    ],
                    target_stds=[
                        0.05,
                        0.05,
                        0.1,
                        0.1,
                    ],
                    type='DeltaXYWHBBoxCoder'),
                fc_out_channels=1024,
                in_channels=256,
                loss_bbox=dict(beta=1.0, loss_weight=1.0,
type='SmoothL1Loss'),
                loss_cls=dict(
                    loss_weight=1.0,
                    type='CrossEntropyLoss',
                    use_sigmoid=False),
                num_classes=14,
                reg_class_agnostic=True,
                roi_feat_size=7,
                type='Shared2FCBBoxHead'),
            dict(
                bbox_coder=dict(
                    target_means=[
                        0.0,
                        0.0,
                        0.0,
                        0.0,
                    ],
                    target_stds=[
                        0.033,
                        0.033,
                        0.067,
                        0.067,
                    ],
                    type='DeltaXYWHBBoxCoder'),
                fc_out_channels=1024,
                in_channels=256,
```

```
                loss_bbox=dict(beta=1.0, loss_weight=1.0,
type='SmoothL1Loss'),
                loss_cls=dict(
                    loss_weight=1.0,
                    type='CrossEntropyLoss',
                    use_sigmoid=False),
                num_classes=14,
                reg_class_agnostic=True,
                roi_feat_size=7,
                type='Shared2FCBBoxHead'),
        ],
        bbox_roi_extractor=dict(
            featmap_strides=[
                4,
                8,
                16,
                32,
            ],
            out_channels=256,
            roi_layer=dict(output_size=7, sampling_ratio=0,
type='RoIAlign'),
            type='SingleRoIExtractor'),
        num_stages=3,
        stage_loss_weights=[
            1,
            0.5,
            0.25,
        ],
        type='CascadeRoIHead'),
    rpn_head=dict(
        anchor_generator=dict(
            ratios=[
                0.5,
                1.0,
                2.0,
            ],
            scales=[
                8,
            ],
            strides=[
                4,
                8,
                16,
                32,
                64,
            ],
            type='AnchorGenerator'),
        bbox_coder=dict(
            target_means=[
                0.0,
                0.0,
                0.0,
                0.0,
            ],
```

```
            target_stds=[
                1.0,
                1.0,
                1.0,
                1.0,
            ],
            type='DeltaXYWHBBoxCoder'),
        feat_channels=256,
        in_channels=256,
        loss_bbox=dict(
            beta=0.1111111111111111, loss_weight=1.0,
type='SmoothL1Loss'),
        loss_cls=dict(
            loss_weight=1.0, type='CrossEntropyLoss', use_sigmoid=True),
        type='RPNHead'),
    test_cfg=dict(
        rcnn=dict(
            max_per_img=100,
            nms=dict(iou_threshold=0.5, type='nms'),
            score_thr=0.05),
        rpn=dict(
            max_per_img=1000,
            min_bbox_size=0,
            nms=dict(iou_threshold=0.7, type='nms'),
            nms_pre=1000)),
    train_cfg=dict(
        rcnn=[
            dict(
                assigner=dict(
                    ignore_iof_thr=-1,
                    match_low_quality=False,
                    min_pos_iou=0.5,
                    neg_iou_thr=0.5,
                    pos_iou_thr=0.5,
                    type='MaxIoUAssigner'),
                debug=False,
                pos_weight=-1,
                sampler=dict(
                    add_gt_as_proposals=True,
                    neg_pos_ub=-1,
                    num=512,
                    pos_fraction=0.25,
                    type='RandomSampler')),
            dict(
                assigner=dict(
                    ignore_iof_thr=-1,
                    match_low_quality=False,
                    min_pos_iou=0.6,
                    neg_iou_thr=0.6,
                    pos_iou_thr=0.6,
                    type='MaxIoUAssigner'),
                debug=False,
                pos_weight=-1,
                sampler=dict(
```

```
                    add_gt_as_proposals=True,
                    neg_pos_ub=-1,
                    num=512,
                    pos_fraction=0.25,
                    type='RandomSampler')),
            dict(
                assigner=dict(
                    ignore_iof_thr=-1,
                    match_low_quality=False,
                    min_pos_iou=0.7,
                    neg_iou_thr=0.7,
                    pos_iou_thr=0.7,
                    type='MaxIoUAssigner'),
                debug=False,
                pos_weight=-1,
                sampler=dict(
                    add_gt_as_proposals=True,
                    neg_pos_ub=-1,
                    num=512,
                    pos_fraction=0.25,
                    type='RandomSampler')),
        ],
        rpn=dict(
            allowed_border=0,
            assigner=dict(
                ignore_iof_thr=-1,
                match_low_quality=True,
                min_pos_iou=0.3,
                neg_iou_thr=0.3,
                pos_iou_thr=0.7,
                type='MaxIoUAssigner'),
            debug=False,
            pos_weight=-1,
            sampler=dict(
                add_gt_as_proposals=False,
                neg_pos_ub=-1,
                num=256,
                pos_fraction=0.5,
                type='RandomSampler')),
        rpn_proposal=dict(
            max_per_img=2000,
            min_bbox_size=0,
            nms=dict(iou_threshold=0.7, type='nms'),
            nms_pre=2000)),
    type='CascadeRCNN')
optim_wrapper = dict(
    clip_grad=None,
    optimizer=dict(lr=0.002, momentum=0.9, type='SGD',
weight_decay=0.0001),
    type='OptimWrapper')
param_scheduler = [
    dict(
        begin=0, by_epoch=False, end=500, start_factor=0.001,
type='LinearLR'),
```

```
    dict(
        begin=0,
        by_epoch=True,
        end=12,
        gamma=0.1,
        milestones=[
            8,
            11,
        ],
        type='MultiStepLR'),
]
resume = False
seed = 0
test_cfg = dict(type='TestLoop')
test_dataloader = dict(
    batch_size=1,
    dataset=dict(
        ann_file='test_json_PATH',
        backend_args=None,
        data_prefix=dict(img='test_img_PATH'),
        data_root='data_PATH',
        metainfo=dict(
            classes=(
                'Atrophy',
                'Stage01',
                'Stage02-03',
                'Stage04',
                'Stage05',
                'Stage06',
                'Stage07',
                'Stage08',
                'Stage09',
                'Stage10',
                'Stage11',
                'Stage12',
                'Stage13',
                'Stage14',
            )),
        pipeline=[
            dict(backend_args=None, type='LoadImageFromFile'),
            dict(keep_ratio=True, scale=(
                2048,
                2048,
            ), type='Resize'),
            dict(
                meta_keys=(
                    'img_id',
                    'img_path',
                    'ori_shape',
                    'img_shape',
                    'scale_factor',
                ),
                type='PackDetInputs'),
        ],
```

```
        test_mode=True,
        type='CocoDataset'),
    drop_last=False,
    num_workers=2,
    persistent_workers=True,
    sampler=dict(shuffle=False, type='DefaultSampler'))
test_evaluator = dict(
    ann_file=
    'test_json_PATH',
    backend_args=None,
    classwise=True,
    format_only=False,
    metric=[
        'bbox',
    ],
    type='CocoMetric')
test_pipeline = [
    dict(backend_args=None, type='LoadImageFromFile'),
    dict(keep_ratio=True, scale=(
        2048,
        2048,
    ), type='Resize'),
    dict(
        meta_keys=(
            'img_id',
            'img_path',
            'ori_shape',
            'img_shape',
            'scale_factor',
        ),
        type='PackDetInputs'),
]
train_cfg = dict(max_epochs=12, type='EpochBasedTrainLoop',
val_interval=1)
train_dataloader = dict(
    batch_sampler=dict(type='AspectRatioBatchSampler'),
    batch_size=2,
    dataset=dict(
        ann_file='train_json_PATH',
        backend_args=None,
        data_prefix=dict(img='train_img_PATH'),
        data_root='data_PATH',
        filter_cfg=dict(filter_empty_gt=True, min_size=32),
        metainfo=dict(
            classes=(
                'Atrophy',
                'Stage01',
                'Stage02-03',
                'Stage04',
                'Stage05',
                'Stage06',
                'Stage07',
                'Stage08',
                'Stage09',
```

```
                'Stage10',
                'Stage11',
                'Stage12',
                'Stage13',
                'Stage14',
            )),
        pipeline=[
            dict(backend_args=None, type='LoadImageFromFile'),
            dict(type='LoadAnnotations', with_bbox=True),
            dict(keep_ratio=True, scale=(
                2048,
                2048,
            ), type='Resize'),
            dict(prob=0.5, type='RandomFlip'),
            dict(type='PackDetInputs'),
        ],
        type='CocoDataset'),
    num_workers=2,
    persistent_workers=True,
    sampler=dict(shuffle=True, type='DefaultSampler'))
train_pipeline = [
    dict(backend_args=None, type='LoadImageFromFile'),
    dict(type='LoadAnnotations', with_bbox=True),
    dict(keep_ratio=True, scale=(
        2048,
        2048,
    ), type='Resize'),
    dict(prob=0.5, type='RandomFlip'),
    dict(type='PackDetInputs'),
]
val_cfg = dict(type='ValLoop')
val_dataloader = dict(
    batch_size=1,
    dataset=dict(
        ann_file='valid_json_PATH',
        backend_args=None,
        data_prefix=dict(img='valid_img_PATH'),
        data_root='data_PATH',
        metainfo=dict(
            classes=(
                'Atrophy',
                'Stage01',
                'Stage02-03',
                'Stage04',
                'Stage05',
                'Stage06',
                'Stage07',
                'Stage08',
                'Stage09',
                'Stage10',
                'Stage11',
                'Stage12',
                'Stage13',
                'Stage14',
```

```
            )),
        pipeline=[
            dict(backend_args=None, type='LoadImageFromFile'),
            dict(keep_ratio=True, scale=(
                2048,
                2048,
            ), type='Resize'),
            dict(
                meta_keys=(
                    'img_id',
                    'img_path',
                    'ori_shape',
                    'img_shape',
                    'scale_factor',
                ),
                type='PackDetInputs'),
        ],
        test_mode=True,
        type='CocoDataset'),
    drop_last=False,
    num_workers=2,
    persistent_workers=True,
    sampler=dict(shuffle=False, type='DefaultSampler'))
val_evaluator = dict(
    ann_file=
    'valid_json_PATH',
    backend_args=None,
    classwise=True,
    format_only=False,
    metric=[
        'bbox',
    ],
    type='CocoMetric')
vis_backends = [
    dict(
        save_dir='save_PATH',
        type='LocalVisBackend'),
    ]
visualizer = dict(
    name='visualizer',
    type='DetLocalVisualizer',
    vis_backends=[
        dict(
            save_dir='save_PATH',
            type='LocalVisBackend'),
    ])
work_dir = 'output_PATH'
```
