# Supplementary material for: Automated spermatogenic staging in periodic acid-Schiff-stained testes of Sprague–Dawley rats using a deep learning model for normal and atrophied tissues
Source: PLoS One. 2026 Jun 29;21(6):e0337245. doi: 10.1371/journal.pone.0337245 (PMC13313349; doi:10.1371/journal.pone.0337245)
Supplement: Attachment — Submitted filename: Plos_one_review.docx [file pone.0337245.s006.docx]

The present study presents a region-based convolutional neural network designed to predict spermatogenic stages from PAS-stained testicular tissue sections. The authors have incorporated robust statistical procedures and report that the model can distinguish 14 stages with an average accuracy of 0.86. Additionally, the framework appears capable of quantitative grading by estimating proportional differences between atrophied and normal seminiferous tubules. Although the manuscript presents the data nicely, the placement of figure descriptions within the main text disrupts the logical flow of the manuscript. All figure legends should be positioned alongside the respective figures or compiled at the end of the discussion section.

Below are a few concerns regarding the model development and interpretation:

**Major concerns**

1. The manuscript does not sufficiently explain how the model mitigates overfitting or handles class imbalance. This issue is particularly relevant because the model was trained using images derived from only a single atrophic slide, raising concerns about the generalizability and robustness of the classifier.
2. As shown in **Figure 4**, while most stages show satisfactory performance, the average precision for stage V remains below 0.7. The authors should provide a justification or discuss potential biological or technical reasons underlying this discrepancy.
3. Given the substantial morphological similarity among spermatogenic stages, developing a multi-class classifier is inherently challenging. Are there any other existing image-based/histology-based models for a similar multi-class classification? The authors should also clarify how a multi-class stage prediction is a better approach than a preliminary binary classification (atrophy vs. normal).

**Minor concerns/Points to add in the discussion**

1. The authors should briefly explain the use of PAS staining over H&E for identifying spermatogenic stages. Adding this clarification will contextualize the methodological choice and help readers, especially those less familiar with testicular histology, better understand the rationale behind the selected staining approach.
2. A clearer explanation of how determining the precise stage of spermatogenesis contributes to clinical decision-making, particularly in infertility diagnostics or testicular pathology, would strengthen the manuscript.
3. **Figure 7** shows differences between the model’s predictions and the literature for stages I, VI, and XII. These discrepancies require further clarification. The authors should elaborate on whether the inconsistencies arise from staining variation, annotation subjectivity, or intrinsic challenges in differentiating these stages.
