# Supplementary material for: Automated spermatogenic staging in periodic acid-Schiff-stained testes of Sprague–Dawley rats using a deep learning model for normal and atrophied tissues
Source: PLoS One. 2026 Jun 29;21(6):e0337245. doi: 10.1371/journal.pone.0337245 (PMC13313349; doi:10.1371/journal.pone.0337245)
Supplement: Attachment — Submitted filename: Response to Reviewers.docx [file pone.0337245.s007.docx]

PONE-D-25-59697

Automated spermatogenic staging in PAS-stained testes of Sprague-Dawley rats using a deep learning model for nomal and atrophied tissues

PLOS One

Dear Dr. Son,

Thank you for submitting your manuscript to PLOS ONE. After careful consideration, we feel that it has merit but does not fully meet PLOS ONE’s publication criteria as it currently stands. Therefore, we invite you to submit a revised version of the manuscript that addresses the points raised during the review process.

While it is an very interesting study, the robustness of the predictions shall be made basing on multiple observations from different slides of atrophied tissues.

We agree with your observation. The revised Discussion explicitly acknowledges that training included a limited number of atrophied specimens, including a single atrophied slide, and discusses the implications for generalizability. The need for validation using larger cohorts with multiple atrophied samples is now clearly stated as a major future direction.

Low precision score in predicting the Stage V should be justified and the possible technical reasons should be described thoroughly

Thank you for the remark. The Discussion now provides a detailed explanation that stage V exhibits broad morphological variability and substantial overlap with adjacent stages due to its wide acrosomal angle range. Technical challenges related to partial or oblique tubule sectioning are also discussed, consistent with known difficulties in manual staging.

Provide details on biological processes in which multi-class staging is carried out and justify its applicability and that it is better than the binary staging process.

We have added a dedicated explanation in the discussion, clarifying that spermatogenesis is a cyclic, stage-specific process and that toxicant effects can target specific stages. Multi-class staging enables localization of disruption along the spermatogenic continuum and provides greater mechanistic insight than binary (normal vs. atrophic) classification.

Provide complete technical details and restrict to the results obtained without overemphasising.

Technical details regarding model design, inference efficiency, and performance trade-offs are now described concisely and in direct relation to the reported results, avoiding overemphasis or speculative claims.

Strong rationale for carrying out this study and justification that is based on previous studies should be included in the introduction. Moreover, the superiority of the assessment made over the existing ones should also be thoroughly presented.

The Introduction has been revised to more clearly position this study relative to prior segmentation-based approaches, highlighting the advantages of an object detection–based framework combined with PAS staining in achieving full stage resolution with improved computational efficiency.

Discussion should be robust and avoid reiterating the observed results. Conclusions should focus on what are the benefits of this study in research and clinical terms and what are the future possibilities for the results presented in this study.

The Discussion has been rewritten to emphasize biological interpretation rather than result reiteration. The Conclusion now explicitly outlines research benefits, practical implications for toxicologic pathology, and future applications.

Please submit your revised manuscript by Mar 01 2026 11:59PM. If you will need more time than this to complete your revisions, please reply to this message or contact the journal office at [plosone@plos.org](mailto:plosone@plos.org). When you're ready to submit your revision, log on to <https://www.editorialmanager.com/pone/> and select the 'Submissions Needing Revision' folder to locate your manuscript file.

Please include the following items when submitting your revised manuscript:

- A letter that responds to each point raised by the academic editor and reviewer(s). You should upload this letter as a separate file labeled 'Response to Reviewers'.
- A marked-up copy of your manuscript that highlights changes made to the original version. You should upload this as a separate file labeled 'Revised Manuscript with Track Changes'.
- An unmarked version of your revised paper without tracked changes. You should upload this as a separate file labeled 'Manuscript'.

If you would like to make changes to your financial disclosure, please include your updated statement in your cover letter. Guidelines for resubmitting your figure files are available below the reviewer comments at the end of this letter.

If applicable, we recommend that you deposit your laboratory protocols in [protocols.io](http://protocols.io/) to enhance the reproducibility of your results. Protocols.io assigns your protocol its own identifier (DOI) so that it can be cited independently in the future. For instructions see: [https://journals.plos.org/plosone/s/submission-guidelines#loc-laboratory-protocols](https://track.editorialmanager.com/CL0/https:%2F%2Fjournals.plos.org%2Fplosone%2Fs%2Fsubmission-guidelines%23loc-laboratory-protocols/1/010f019bc4936d0a-2d592588-850f-4400-aa66-61b05aa7c2cc-000000/eD6yFn8LOLF9UQ1oHFzBetAqSyFjwfquRrYnO1pbWrY=246). Additionally, PLOS ONE offers an option for publishing peer-reviewed Lab Protocol articles, which describe protocols hosted on [protocols.io](http://protocols.io/). Read more information on sharing protocols at [https://plos.org/protocols?utm_medium=editorial-email&utm_source=authorletters&utm_campaign=protocols](https://track.editorialmanager.com/CL0/https:%2F%2Fplos.org%2Fprotocols%3Futm_medium=editorial-email%26utm_source=authorletters%26utm_campaign=protocols/1/010f019bc4936d0a-2d592588-850f-4400-aa66-61b05aa7c2cc-000000/z6Ncy_gmH7-q3464Ea2lk8d8CRRcRskNSC7awOzFppk=246).

We look forward to receiving your revised manuscript.

Kind regards,

Suresh Yenugu
Academic Editor
PLOS One

Journal Requirements:

When submitting your revision, we need you to address these additional requirements.

1. Please ensure that your manuscript meets PLOS ONE's style requirements, including those for file naming. The PLOS ONE style templates can be found at

<https://journals.plos.org/plosone/s/file?id=wjVg/PLOSOne_formatting_sample_main_body.pdf> and
[https://journals.plos.org/plosone/s/file?id=ba62/PLOSOne_formatting_sample_title_authors_affiliations.pdf](https://track.editorialmanager.com/CL0/https:%2F%2Fjournals.plos.org%2Fplosone%2Fs%2Ffile%3Fid=ba62%2FPLOSOne_formatting_sample_title_authors_affiliations.pdf/1/010f019bc4936d0a-2d592588-850f-4400-aa66-61b05aa7c2cc-000000/swxL0O-4ldlFg4G1dN4wggA7rT-oCYpKLuveB7dFQoU=246)

We have reviewed the manuscript and confirmed that it meets the PLOS ONE style requirements, including file naming conventions.

2. To comply with PLOS One submissions requirements, in your Methods section, please provide additional information regarding the experiments involving animals and ensure you have included details on (1) methods of sacrifice, (2) methods of anesthesia and/or analgesia, and (3) efforts to alleviate suffering.

The requested information regarding animal experiments, including methods of sacrifice, anesthesia/analgesia, and efforts to alleviate suffering, has been added to the Methods section.

3. Please note that your Data Availability Statement is currently missing the repository name. If your manuscript is accepted for publication, you will be asked to provide these details on a very short timeline. We therefore suggest that you provide this information now, though we will not hold up the peer review process if you are unable.

The repository name (Figshare) has been added to the Data Availability Statement.

4. Thank you for stating the following in the Competing Interests section:

[The authors have declared that no competing interests exist.].

We note that one or more of the authors are employed by a commercial company: CorestemChemon Nonclinical Research Institute

1. Please provide an amended Funding Statement declaring this commercial affiliation, as well as a statement regarding the Role of Funders in your study. If the funding organization did not play a role in the study design, data collection and analysis, decision to publish, or preparation of the manuscript and only provided financial support in the form of authors' salaries and/or research materials, please review your statements relating to the author contributions, and ensure you have specifically and accurately indicated the role(s) that these authors had in your study. You can update author roles in the Author Contributions section of the online submission form.

Please also include the following statement within your amended Funding Statement.

“The funder provided support in the form of salaries for authors [insert relevant initials], but did not have any additional role in the study design, data collection and analysis, decision to publish, or preparation of the manuscript. The specific roles of these authors are articulated in the ‘author contributions’ section.”

If your commercial affiliation did play a role in your study, please state and explain this role within your updated Funding Statement.

2. Please also provide an updated Competing Interests Statement declaring this commercial affiliation along with any other relevant declarations relating to employment, consultancy, patents, products in development, or marketed products, etc.

Within your Competing Interests Statement, please confirm that this commercial affiliation does not alter your adherence to all PLOS ONE policies on sharing data and materials by including the following statement: "This does not alter our adherence to PLOS ONE policies on sharing data and materials.” (as detailed online in our guide for authors <http://journals.plos.org/plosone/s/competing-interests>) . If this adherence statement is not accurate and there are restrictions on sharing of data and/or materials, please state these. Please note that we cannot proceed with consideration of your article until this information has been declared.

Please include both an updated Funding Statement and Competing Interests Statement in your cover letter. We will change the online submission form on your behalf.

The Funding Statement and Competing Interests Statement have been updated accordingly.

5. Please include your full ethics statement in the ‘Methods’ section of your manuscript file. In your statement, please include the full name of the IRB or ethics committee who approved or waived your study, as well as whether or not you obtained informed written or verbal consent. If consent was waived for your study, please include this information in your statement as well.

The full ethics statement has been added to the Methods section, including the name of the ethics committee and consent information.

6. Please upload a new copy of Figures 5, 8, 9, 10, S1, and S2 as the details are not clear. Please follow the link for more information:  [https://journals.plos.org/plosone/s/figures](https://track.editorialmanager.com/CL0/https:%2F%2Fjournals.plos.org%2Fplosone%2Fs%2Ffigures/1/010f019bc4936d0a-2d592588-850f-4400-aa66-61b05aa7c2cc-000000/0GuceG3Yox9ieQaUkWbjwWxIxlwpJIt9xSd9VbgYGgw=246)

To improve figure clarity while maintaining manageable file sizes, representative images were selected and partially enlarged to better display the relevant details.

7. Please ensure that you refer to Table S1 in your text as, if accepted, production will need this reference to link the reader to the table.

A reference to Table S1 has been added to the main text.

8. Please include captions for your Supporting Information files at the end of your manuscript (S4_table_old.pdf), and update any in-text citations to match accordingly. Please see our Supporting Information guidelines for more information: <http://journals.plos.org/plosone/s/supporting-information>.

The file S4_table_old.pdf was removed because it is no longer used.

If the reviewer comments include a recommendation to cite specific previously published works, please review and evaluate these publications to determine whether they are relevant and should be cited. There is no requirement to cite these works unless the editor has indicated otherwise. 


[Note: HTML markup is below. Please do not edit.]

Reviewers' comments:

Reviewer's Responses to Questions

**Comments to the Author**

1. Is the manuscript technically sound, and do the data support the conclusions?

The manuscript must describe a technically sound piece of scientific research with data that supports the conclusions. Experiments must have been conducted rigorously, with appropriate controls, replication, and sample sizes. The conclusions must be drawn appropriately based on the data presented.

Reviewer #1: Partly

Reviewer #2: Partly

2. Has the statistical analysis been performed appropriately and rigorously?

Reviewer #1: Yes

Reviewer #2: Yes

3. Have the authors made all data underlying the findings in their manuscript fully available?

The [PLOS Data policy](http://track.editorialmanager.com/CL0/http:%2F%2Fwww.plosone.org%2Fstatic%2Fpolicies.action%23sharing/1/010f019bc4936d0a-2d592588-850f-4400-aa66-61b05aa7c2cc-000000/8NflRu2pJkodHrwS9upHeaB3tbON9W5GCsuVIrSzUv4=246) requires authors to make all data underlying the findings described in their manuscript fully available without restriction, with rare exception (please refer to the Data Availability Statement in the manuscript PDF file). The data should be provided as part of the manuscript or its supporting information, or deposited to a public repository. For example, in addition to summary statistics, the data points behind means, medians and variance measures should be available. If there are restrictions on publicly sharing data—e.g. participant privacy or use of data from a third party—those must be specified.

Reviewer #1: Yes

Reviewer #2: Yes

4. Is the manuscript presented in an intelligible fashion and written in standard English?

PLOS ONE does not copyedit accepted manuscripts, so the language in submitted articles must be clear, correct, and unambiguous. Any typographical or grammatical errors should be corrected at revision, so please note any specific errors here.

Reviewer #1: Yes

Reviewer #2: No

5. Review Comments to the Author

Please use the space provided to explain your answers to the questions above. You may also include additional comments for the author, including concerns about dual publication, research ethics, or publication ethics. (Please upload your review as an attachment if it exceeds 20,000 characters)

Reviewer #1: The present study presents a region-based convolutional neural network designed to predict spermatogenic stages from PAS-stained testicular tissue sections. The authors have incorporated robust statistical procedures and report that the model can distinguish 14 stages with an average accuracy of 0.86. Additionally, the framework appears capable of quantitative grading by estimating proportional differences between atrophied and normal seminiferous tubules. Although the manuscript presents the data nicely, the placement of figure descriptions within the main text disrupts the logical flow of the manuscript. All figure legends should be positioned alongside the respective figures or compiled at the end of the discussion section.
Below are a few concerns regarding the model development and interpretation:
Major concerns
1. The manuscript does not sufficiently explain how the model mitigates overfitting or handles class imbalance. This issue is particularly relevant because the model was trained using images derived from only a single atrophic slide, raising concerns about the generalizability and robustness of the classifier.

In the Discussion, we have clarified how overfitting and class imbalance were addressed in the model, including the use of data augmentation applied across all stages and the intrinsic balancing effect of the Cascade R-CNN architecture. While the training data were derived from a single atrophic slide, the model was trained on a large number of morphologically diverse seminiferous tubules, which partially mitigates overfitting at the tubule level.

2. As shown in Figure 4, while most stages show satisfactory performance, the average precision for stage V remains below 0.7. The authors should provide a justification or discuss potential biological or technical reasons underlying this discrepancy.

This point is now addressed in detail in the Discussion. We explain that the lower AP for stage V reflects intrinsic biological variability and technical challenges in acrosomal angle assessment rather than model instability. This explanation is supported by established spermatogenic staging criteria and parallels known limitations in manual evaluation.

3. Given the substantial morphological similarity among spermatogenic stages, developing a multi-class classifier is inherently challenging. Are there any other existing image-based/histology-based models for a similar multi-class classification? The authors should also clarify how a multi-class stage prediction is a better approach than a preliminary binary classification (atrophy vs. normal).

We acknowledge your points. We explained in the Discussion why a multi-class prediction was chosen. Where a binary atrophy or normal classifier would detect gross abnormalities, our multi-class model could differentiate subtle stage-specific patterns, which is important for understanding progression and pathology.

Minor concerns/Points to add in the discussion
1. The authors should briefly explain the use of PAS staining over H&E for identifying spermatogenic stages. Adding this clarification will contextualize the methodological choice and help readers, especially those less familiar with testicular histology, better understand the rationale behind the selected staining approach.

A brief explanation has been added to the Introduction and Discussion, noting that PAS staining enhances visualization of the spermatid acrosome, which is essential for reliable discrimination of spermatogenic stages.

2. A clearer explanation of how determining the precise stage of spermatogenesis contributes to clinical decision-making, particularly in infertility diagnostics or testicular pathology, would strengthen the manuscript.

We have revised the Discussion to explicitly explain how precise determination of spermatogenic stages, in the context of a multi-class staging framework, contributes to clinical decision-making.

3. Figure 7 shows differences between the model’s predictions and the literature for stages I, VI, and XII. These discrepancies require further clarification. The authors should elaborate on whether the inconsistencies arise from staining variation, annotation subjectivity, or intrinsic challenges in differentiating these stages.

We have expanded the Discussion to explain that these discrepancies are unlikely to reflect systematic misclassification, as no pronounced reciprocal shifts between adjacent stages were observed. Instead, potential contributors such as biological variability, limited animal numbers, staining variation, and annotation subjectivity are discussed.

Reviewer #2: The manuscript addresses the application of deep learning–based object detection for automated spermatogenic stage classification and atrophy detection in rat testes. While the study presents promising results, several aspects require clarification and improvement, particularly regarding dataset size, methodological transparency, statistical analysis, and interpretation of results.

Abstract
1.The abstract overemphasizes model performance without sufficiently contextualizing the limited sample size.

We appreciate your comment. We agree that the limited sample size should be more clearly contextualized. Accordingly, we have revised the abstract to explicitly state the number of animals and whole-slide images analyzed and to emphasize that the reported performance primarily demonstrates the feasibility of the proposed approach rather than full generalizability.

2.The number of animals and WSIs analyzed should be explicitly stated.

We have revised the abstract to explicitly state that a total of 16 WSIs obtained from 16 Sprague-Dawley rats were included in the study, encompassing both normal and atrophied testicular samples.

3.The term “atrophy grading” should be clarified as quantitative detection/counting rather than conventional histopathological grading.

Thank you for the clarification. The term “atrophy grading” has been revised as such.

4.Questions:
- How many rats and WSIs were included in the training, validation, and test datasets

We have clarified the dataset composition in the abstract and Methods section. Specifically, 10 WSIs (9 normal and 1 atrophied testes) were used for model training, validation, and testing, while the remaining 6 WSIs (3 normal and 3 atrophied testes) were used for inference

- Were atrophied samples included during model training or used only for inference?

Atrophied samples were included in limited numbers during training to enable the model to learn the morphological features of atrophied seminiferous tubules. Additional atrophied WSIs were excluded from training and used exclusively for inference to evaluate the applicability of the model to real-world pathological conditions.

Introduction
1.The novelty of this study relative to previous AI-based spermatogenic staging studies is not clearly articulated.

The novelty of this study lies in (1) the use of a two-stage object detection framework for direct seminiferous tubule-level stage classification without pixel-level segmentation, (2) the application of PAS-stained WSIs, enabling discrimination of all 14 spermatogenic stages without stage grouping, and (3) the inclusion of atrophied tubules with a practical demonstration of lesion severity grading. To our knowledge, these elements have not been jointly addressed in previous AI-based spermatogenic staging studies.

2.The rationale for selecting an object detection framework (R-CNN family) over segmentation or classification-based approaches requires stronger justification.

Object detection was selected because spermatogenic stage determination relies on global tubule-level morphology rather than precise pixel-level boundaries. Segmentation-based pipelines are computationally intensive and prone to error propagation across multiple steps. In contrast, object detection enables efficient tubule localization and direct stage classification, which is sufficient for stage frequency analysis and large-scale WSI inference. A two-stage R-CNN framework was chosen for its higher accuracy in complex histological images.

3.Prior studies using deep learning for testicular histopathology should be discussed in more depth to better position the contribution of this work.

Prior studies predominantly used U-Net–based segmentation, followed by rule-based or decision tree classifiers, and were mostly limited to H&E-stained images, resulting in grouped stage classifications. We have clarified these differences in the manuscript to better position the contribution of our detector-based, PAS-driven approach.

4.Questions:
- What specific limitations of existing automated spermatogenic staging methods does this study aim to overcome?

This study addresses the high computational cost, error accumulation in multi-step segmentation pipelines, limited stage resolution due to H&E staining, and lack of applicability to pathological conditions in existing automated spermatogenic staging methods.

- Why was object detection considered more suitable than instance or semantic segmentation for this task?
Pixel-level segmentation provides limited additional value for stage determination while increasing computational burden. Object detection offers a more efficient and scalable solution by capturing biologically relevant tubule-level features required for spermatogenic staging.

Materials and Methods
1.The small dataset size (number of animals, WSIs, and annotated tubules) should be more explicitly described and discussed.

We acknowledge this limitation and have clarified the number of animals, WSIs, and annotated seminiferous tubules in the revised manuscript. We also explicitly discuss the limited animal number as a constraint on animal-level generalizability, while noting that the large number of tubules per WSI enables robust tubule-level inference.

2.Details regarding annotation procedures, consensus building, and inter-pathologist variability are insufficient.

To ensure annotation consistency, all annotations were performed by a single pathologist following predefined staging criteria, and the annotations were reviewed multiple times for internal consistency. We have clarified this procedure in the Methods section.

3.The data split strategy should be clarified to ensure that data from the same animal were not used across training and test sets.

As now clarified in the Methods section, the dataset was partitioned at the tile level, and tiles derived from the same animal could therefore be included across the training, validation, and test datasets. This strategy was adopted to increase the number of training samples, given the limited number of animals. We acknowledge that this split does not fully assess animal-level generalization and have explicitly stated this as a limitation. Future studies with larger cohorts will employ animal-level data partitioning to further validate model robustness.

4. The statistical methods used for stage frequency comparisons require a clearer description, including assumptions and multiple comparison handling.

We thank the reviewer for this important comment. The Statistical Analysis section has been revised to provide a clearer description of the analytical framework. Specifically, we now clarify that sample-level stage proportions were used for group comparisons and that MANOVA was applied in an exploratory manner to evaluate overall differences in stage-wise distributions, under the assumptions of approximate multivariate normality and homogeneity of covariance matrices. When a significant multivariate effect was observed, Dunnett’s post hoc test was performed with the model results serving as the reference group.

5.Questions:
- How was annotation consistency ensured among pathologists?

To ensure consistency, all annotations were performed by a single pathologist based on predefined criteria and underwent multiple rounds of review to maintain internal consistency.

- Were WSIs from the same rat separated across training, validation, and testing datasets?

This point has been addressed in our response to Comment 3 and clarified in the Methods section. Briefly, data partitioning was performed at the tile level; therefore, tiles derived from the same animal could be included across different datasets. This limitation is now explicitly acknowledged in the revised manuscript.

- How was class imbalance among the 14 spermatogenic stages addressed during training?

We acknowledge the presence of class imbalance among the 14 spermatogenic stages. Although no explicit class reweighting or resampling strategy was applied due to the retrospective nature of the dataset, the effects of imbalance were partially mitigated by the two-stage detection framework. Specifically, the Cascade R-CNN architecture progressively emphasizes harder RoIs at later stages, and a fixed positive-to-negative ratio was enforced during RoI sampling. In addition, model performance was assessed using per-class evaluation metrics to avoid bias toward majority stages. This limitation is now explicitly acknowledged in the revised manuscript.


Results
1.The relatively high mAR combined with lower stage-wise AP for certain stages suggests increased false positives and should be discussed quantitatively.

We now explicitly interpret this pattern as a sensitivity-specificity trade-off, reflecting increased false-positive stage assignments rather than failure to detect seminiferous tubules. Most false positives correspond to correctly localized tubules with incorrect stage labels or duplicate detections, which is now clarified in the Discussion.

2.Lower performance for stages II–III, V, and XI warrants deeper analysis beyond morphological similarity.

The Discussion has been expanded to attribute reduced performance for these stages to stage-specific biological and anatomical factors, including transitional acrosomal development, wide acrosomal angle ranges, short stage duration, and intratubular heterogeneity, rather than technical instability.

3.The reported WSI inference time (~211 seconds) should be discussed in terms of feasibility for routine or large-scale toxicological studies.

The WSI inference time is now discussed in the context of routine and large-scale non-clinical toxicology studies, demonstrating practical feasibility compared with manual staging.

4.Questions:
- Were confidence thresholds optimized for WSI-level inference?

A fixed confidence threshold determined during training was applied to all WSIs; thresholds were not optimized at the WSI level.

- How consistent was model performance across individual animals?

Agreement rates ranged from 90.0% to 93.1% across three independently inferred WSIs, indicating stable performance across animals; detailed per-animal agreement rates have now been added to the Results section.

-Was model robustness evaluated against staining or sectioning variability?

Dedicated robustness experiments were not performed; however, training and inference on PAS-stained WSIs from different specimens inherently incorporated routine histological variability, with stable performance observed.


Discussion
1.The discussion partially reiterates results rather than critically interpreting them.

The Discussion has been revised to reduce repetition of results and emphasize critical biological and toxicological interpretation.

2.The limited number of animals (especially for WSI-level analysis) should be more explicitly acknowledged as a major limitation.

The limited number of animals is now explicitly identified as a major limitation, and the implications for non-clinical regulatory toxicology are discussed with appropriate caution.

3.The implications for regulatory toxicology and standard non-clinical studies require clearer contextualization.

The Discussion has been revised. We emphasize that stage-resolved, whole-slide–level quantification enables objective, reproducible detection of relative and stage-specific changes between experimental groups, which is often more informative for regulatory decision-making than strict agreement with historical absolute stage frequencies.

4.Questions:
- Could data augmentation or balanced sampling strategies improve performance for short-duration stages?

Data augmentation was applied uniformly across all stages, including short-duration stages. However, we acknowledge that image-level augmentation alone may be insufficient to fully capture the intrinsic biological variability of these stages. Explicit balanced sampling was not performed, as the Cascade R-CNN architecture inherently mitigates class imbalance through multi-stage proposal refinement and IoU-based supervision. Nevertheless, stage-aware sampling or loss re-weighting strategies may further improve performance and represent a direction for future work.

- How generalizable is the model to different ages, strains, or staining protocols?

The model was trained on a single age group, strain, and staining protocol, and its generalizability beyond these conditions has not yet been systematically evaluated. While data augmentation may provide limited robustness, validation using external datasets encompassing different ages, strains, and staining protocols will be necessary to confirm broader applicability.

6. Conclusion
1. The conclusion is overly long and contains repetitive statements already addressed in the Discussion.

We have condensed the Conclusion to eliminate redundancy and to focus exclusively on the core findings and their implications.

2. It should be condensed to focus on the principal findings and their implications.

The revised Conclusion has been streamlined to emphasize the central outcome of this study:

3. Claims regarding “objective standards” should be tempered given the limited dataset size.

We appreciate your comment. In the revised text, we have tempered language implying objective or definitive standards and instead describe the framework as a “scalable and supportive” tool.

4. Questions
- Can the authors explicitly state the main scientific contribution and the main practical contribution of this study?

To directly address this point, we now explicitly distinguish the two main contributions of this study in the Conclusion:

- Main scientific contribution: The study demonstrates that spermatogenic stages can be quantitatively analyzed across entire testes using an object detection-based approach, enabling stage-resolved frequency analysis and detection of subtle, stage-specific perturbations relevant to mechanistic reproductive toxicology.
- Main practical contribution: The proposed framework provides automated tubule-level stage classification and atrophy quantification, reducing manual workload and inter-observer variability while improving consistency in routine histopathological evaluation.

6. PLOS authors have the option to publish the peer review history of their article ([what does this mean?](https://track.editorialmanager.com/CL0/https:%2F%2Fjournals.plos.org%2Fplosone%2Fs%2Feditorial-and-peer-review-process%23loc-peer-review-history/1/010f019bc4936d0a-2d592588-850f-4400-aa66-61b05aa7c2cc-000000/1EX0Sbcplri33cCDEfwFCBEs-1GbpoT8icJp8V5WZAo=246)). If published, this will include your full peer review and any attached files.

If you choose “no”, your identity will remain anonymous but your review may still be made public.

**Do you want your identity to be public for this peer review?** For information about this choice, including consent withdrawal, please see our [Privacy Policy](https://track.editorialmanager.com/CL0/https:%2F%2Fwww.plos.org%2Fprivacy-policy/1/010f019bc4936d0a-2d592588-850f-4400-aa66-61b05aa7c2cc-000000/BTGcMHEAIvs1L1i1xMsykPHwL7O2gSgDvV1ofWYyOeE=246).

Reviewer #1: No

Reviewer #2: No

[NOTE: If reviewer comments were submitted as an attachment file, they will be attached to this email and accessible via the submission site. Please log into your account, locate the manuscript record, and check for the action link "View Attachments". If this link does not appear, there are no attachment files.]

To ensure your figures meet our technical requirements, please review our figure guidelines: <https://journals.plos.org/plosone/s/figures>

You may also use PLOS’s free figure tool, NAAS, to help you prepare publication quality figures: <https://journals.plos.org/plosone/s/figures#loc-tools-for-figure-preparation>.

NAAS will assess whether your figures meet our technical requirements by comparing each figure against our figure specifications.

*In compliance with data protection regulations, you may request that we remove your personal registration details at any time.* [*(Remove my information/details)*](https://track.editorialmanager.com/CL0/https:%2F%2Fwww.editorialmanager.com%2Fpone%2Flogin.asp%3Fa=r/1/010f019bc4936d0a-2d592588-850f-4400-aa66-61b05aa7c2cc-000000/Y5PEb2YgYGiB54N9w3GJZXO2pvU1ySxnSRwUza7aniI=246)*. Please contact the publication office if you have any questions.*
