# Supplementary material for: Automated spermatogenic staging in periodic acid-Schiff-stained testes of Sprague–Dawley rats using a deep learning model for normal and atrophied tissues
Source: PLoS One. 2026 Jun 29;21(6):e0337245. doi: 10.1371/journal.pone.0337245 (PMC13313349; doi:10.1371/journal.pone.0337245)
Supplement: Peer review history

# Peer review: Decision Letter 0

**DOI:** 10.1371/journal.pone.0337245.r001

**Authors:** Suresh Yenugu

15 Jan 2026

PONE-D-25-59697

Automated spermatogenic staging in PAS-stained testes of Sprague-Dawley rats using a deep learning model for nomal and atrophied tissues

PLOS One

Dear Dr. Son,

Thank you for submitting your manuscript to PLOS ONE. After careful consideration, we feel that it has merit but does not fully meet PLOS ONE’s publication criteria as it currently stands. Therefore, we invite you to submit a revised version of the manuscript that addresses the points raised during the review process.

While it is an very interesting study, the robustness of the predictions shall be made basing on multiple observations from different slides of atrophied tissues.

Low precision score in predicting the Stage V should be justified and the possible technical reasons should be described thoroughly

Provide details on biological processes in which multi-class staging is carried out and justify its applicability and that it is better than the binary staging process.

Provide complete technical details and restrict to the results obtained without overemphasising.

Strong rationale for carrying out this study and justification that is based on previous studies should be included in the introduction. Moreover, the superiority of the assessment made over the existing ones should also be thoroughly presented.

Discussion should be robust and avoid reiterating the observed results. Conclusions should focus on what are the benefits of this study in research and clinical terms and what are the future possibilities for the results presented in this study.

Please submit your revised manuscript by Mar 01 2026 11:59PM. If you will need more time than this to complete your revisions, please reply to this message or contact the journal office at plosone@plos.org. When you're ready to submit your revision, log on to https://www.editorialmanager.com/pone/ and select the 'Submissions Needing Revision' folder to locate your manuscript file.

Please include the following items when submitting your revised manuscript:

- A letter that responds to each point raised by the academic editor and reviewer(s). You should upload this letter as a separate file labeled 'Response to Reviewers'.
- A marked-up copy of your manuscript that highlights changes made to the original version. You should upload this as a separate file labeled 'Revised Manuscript with Track Changes'.
- An unmarked version of your revised paper without tracked changes. You should upload this as a separate file labeled 'Manuscript'.

If you would like to make changes to your financial disclosure, please include your updated statement in your cover letter. Guidelines for resubmitting your figure files are available below the reviewer comments at the end of this letter.

If applicable, we recommend that you deposit your laboratory protocols in protocols.io to enhance the reproducibility of your results. Protocols.io assigns your protocol its own identifier (DOI) so that it can be cited independently in the future. For instructions see: https://journals.plos.org/plosone/s/submission-guidelines#loc-laboratory-protocols. Additionally, PLOS ONE offers an option for publishing peer-reviewed Lab Protocol articles, which describe protocols hosted on protocols.io. Read more information on sharing protocols at https://plos.org/protocols?utm_medium=editorial-email&utm_source=authorletters&utm_campaign=protocols.

We look forward to receiving your revised manuscript.

Kind regards,

Suresh Yenugu

Academic Editor

PLOS One

Journal Requirements:

When submitting your revision, we need you to address these additional requirements.

1. Please ensure that your manuscript meets PLOS ONE's style requirements, including those for file naming. The PLOS ONE style templates can be found at

https://journals.plos.org/plosone/s/file?id=wjVg/PLOSOne_formatting_sample_main_body.pdf and

https://journals.plos.org/plosone/s/file?id=ba62/PLOSOne_formatting_sample_title_authors_affiliations.pdf

2. To comply with PLOS One submissions requirements, in your Methods section, please provide additional information regarding the experiments involving animals and ensure you have included details on (1) methods of sacrifice, (2) methods of anesthesia and/or analgesia, and (3) efforts to alleviate suffering.

3. Please note that your Data Availability Statement is currently missing the repository name. If your manuscript is accepted for publication, you will be asked to provide these details on a very short timeline. We therefore suggest that you provide this information now, though we will not hold up the peer review process if you are unable.

4. Thank you for stating the following in the Competing Interests section:

[The authors have declared that no competing interests exist.].

We note that one or more of the authors are employed by a commercial company: CorestemChemon Nonclinical Research Institute

1. Please provide an amended Funding Statement declaring this commercial affiliation, as well as a statement regarding the Role of Funders in your study. If the funding organization did not play a role in the study design, data collection and analysis, decision to publish, or preparation of the manuscript and only provided financial support in the form of authors' salaries and/or research materials, please review your statements relating to the author contributions, and ensure you have specifically and accurately indicated the role(s) that these authors had in your study. You can update author roles in the Author Contributions section of the online submission form.

Please also include the following statement within your amended Funding Statement.

“The funder provided support in the form of salaries for authors [insert relevant initials], but did not have any additional role in the study design, data collection and analysis, decision to publish, or preparation of the manuscript. The specific roles of these authors are articulated in the ‘author contributions’ section.”

If your commercial affiliation did play a role in your study, please state and explain this role within your updated Funding Statement.

2. Please also provide an updated Competing Interests Statement declaring this commercial affiliation along with any other relevant declarations relating to employment, consultancy, patents, products in development, or marketed products, etc.

Within your Competing Interests Statement, please confirm that this commercial affiliation does not alter your adherence to all PLOS ONE policies on sharing data and materials by including the following statement: "This does not alter our adherence to PLOS ONE policies on sharing data and materials.” (as detailed online in our guide for authors http://journals.plos.org/plosone/s/competing-interests) . If this adherence statement is not accurate and there are restrictions on sharing of data and/or materials, please state these. Please note that we cannot proceed with consideration of your article until this information has been declared.

Please include both an updated Funding Statement and Competing Interests Statement in your cover letter. We will change the online submission form on your behalf.

5. Please include your full ethics statement in the ‘Methods’ section of your manuscript file. In your statement, please include the full name of the IRB or ethics committee who approved or waived your study, as well as whether or not you obtained informed written or verbal consent. If consent was waived for your study, please include this information in your statement as well.

6. Please upload a new copy of Figures 5, 8, 9, 10, S1, and S2 as the details are not clear. Please follow the link for more information:  https://journals.plos.org/plosone/s/figures

7. Please ensure that you refer to Table S1 in your text as, if accepted, production will need this reference to link the reader to the table.

8. Please include captions for your Supporting Information files at the end of your manuscript (S4_table_old.pdf), and update any in-text citations to match accordingly. Please see our Supporting Information guidelines for more information: http://journals.plos.org/plosone/s/supporting-information.

If the reviewer comments include a recommendation to cite specific previously published works, please review and evaluate these publications to determine whether they are relevant and should be cited. There is no requirement to cite these works unless the editor has indicated otherwise.

[Note: HTML markup is below. Please do not edit.]

Reviewers' comments:

Reviewer's Responses to Questions

**Comments to the Author**

1. Is the manuscript technically sound, and do the data support the conclusions?

The manuscript must describe a technically sound piece of scientific research with data that supports the conclusions. Experiments must have been conducted rigorously, with appropriate controls, replication, and sample sizes. The conclusions must be drawn appropriately based on the data presented.

Reviewer #1: Partly

Reviewer #2: Partly

**********

2. Has the statistical analysis been performed appropriately and rigorously?

Reviewer #1: Yes

Reviewer #2: Yes

**********

3. Have the authors made all data underlying the findings in their manuscript fully available?

The PLOS Data policy requires authors to make all data underlying the findings described in their manuscript fully available without restriction, with rare exception (please refer to the Data Availability Statement in the manuscript PDF file). The data should be provided as part of the manuscript or its supporting information, or deposited to a public repository. For example, in addition to summary statistics, the data points behind means, medians and variance measures should be available. If there are restrictions on publicly sharing data—e.g. participant privacy or use of data from a third party—those must be specified.

Reviewer #1: Yes

Reviewer #2: Yes

**********

4. Is the manuscript presented in an intelligible fashion and written in standard English?

PLOS ONE does not copyedit accepted manuscripts, so the language in submitted articles must be clear, correct, and unambiguous. Any typographical or grammatical errors should be corrected at revision, so please note any specific errors here.

Reviewer #1: Yes

Reviewer #2: No

**********

5. Review Comments to the Author

Please use the space provided to explain your answers to the questions above. You may also include additional comments for the author, including concerns about dual publication, research ethics, or publication ethics. (Please upload your review as an attachment if it exceeds 20,000 characters)

Reviewer #1: The present study presents a region-based convolutional neural network designed to predict spermatogenic stages from PAS-stained testicular tissue sections. The authors have incorporated robust statistical procedures and report that the model can distinguish 14 stages with an average accuracy of 0.86. Additionally, the framework appears capable of quantitative grading by estimating proportional differences between atrophied and normal seminiferous tubules. Although the manuscript presents the data nicely, the placement of figure descriptions within the main text disrupts the logical flow of the manuscript. All figure legends should be positioned alongside the respective figures or compiled at the end of the discussion section.

Below are a few concerns regarding the model development and interpretation:

Major concerns

1. The manuscript does not sufficiently explain how the model mitigates overfitting or handles class imbalance. This issue is particularly relevant because the model was trained using images derived from only a single atrophic slide, raising concerns about the generalizability and robustness of the classifier.

2. As shown in Figure 4, while most stages show satisfactory performance, the average precision for stage V remains below 0.7. The authors should provide a justification or discuss potential biological or technical reasons underlying this discrepancy.

3. Given the substantial morphological similarity among spermatogenic stages, developing a multi-class classifier is inherently challenging. Are there any other existing image-based/histology-based models for a similar multi-class classification? The authors should also clarify how a multi-class stage prediction is a better approach than a preliminary binary classification (atrophy vs. normal).

Minor concerns/Points to add in the discussion

1. The authors should briefly explain the use of PAS staining over H&E for identifying spermatogenic stages. Adding this clarification will contextualize the methodological choice and help readers, especially those less familiar with testicular histology, better understand the rationale behind the selected staining approach.

2. A clearer explanation of how determining the precise stage of spermatogenesis contributes to clinical decision-making, particularly in infertility diagnostics or testicular pathology, would strengthen the manuscript.

3. Figure 7 shows differences between the model’s predictions and the literature for stages I, VI, and XII. These discrepancies require further clarification. The authors should elaborate on whether the inconsistencies arise from staining variation, annotation subjectivity, or intrinsic challenges in differentiating these stages.

Reviewer #2: The manuscript addresses the application of deep learning–based object detection for automated spermatogenic stage classification and atrophy detection in rat testes. While the study presents promising results, several aspects require clarification and improvement, particularly regarding dataset size, methodological transparency, statistical analysis, and interpretation of results.

Abstract

1.The abstract overemphasizes model performance without sufficiently contextualizing the limited sample size.

2.The number of animals and WSIs analyzed should be explicitly stated.

3.The term “atrophy grading” should be clarified as quantitative detection/counting rather than conventional histopathological grading.

4.Questions:

- How many rats and WSIs were included in the training, validation, and test datasets

- Were atrophied samples included during model training or used only for inference?

Introduction

1.The novelty of this study relative to previous AI-based spermatogenic staging studies is not clearly articulated.

2.The rationale for selecting an object detection framework (R-CNN family) over segmentation or classification-based approaches requires stronger justification.

3.Prior studies using deep learning for testicular histopathology should be discussed in more depth to better position the contribution of this work.

4.Questions:

- What specific limitations of existing automated spermatogenic staging methods does this study aim to overcome?

- Why was object detection considered more suitable than instance or semantic segmentation for this task?

Materials and Methods

1.The small dataset size (number of animals, WSIs, and annotated tubules) should be more explicitly described and discussed.

2.Details regarding annotation procedures, consensus building, and inter-pathologist variability are insufficient.

3.The data split strategy should be clarified to ensure that data from the same animal were not used across training and test sets.

4. The statistical methods used for stage frequency comparisons require a clearer description, including assumptions and multiple comparison handling.

5.Questions:

- How was annotation consistency ensured among pathologists?

- Were WSIs from the same rat separated across training, validation, and testing datasets?

- How was class imbalance among the 14 spermatogenic stages addressed during training?

Results

1.The relatively high mAR combined with lower stage-wise AP for certain stages suggests increased false positives and should be discussed quantitatively.

2.Lower performance for stages II–III, V, and XI warrants deeper analysis beyond morphological similarity.

3.The reported WSI inference time (~211 seconds) should be discussed in terms of feasibility for routine or large-scale toxicological studies.

4.Questions:

- Were confidence thresholds optimized for WSI-level inference?

- How consistent was model performance across individual animals?

-Was model robustness evaluated against staining or sectioning variability?

Discussion

1.The discussion partially reiterates results rather than critically interpreting them.

2.The limited number of animals (especially for WSI-level analysis) should be more explicitly acknowledged as a major limitation.

3.The implications for regulatory toxicology and standard non-clinical studies require clearer contextualization.

4.Questions:

- Could data augmentation or balanced sampling strategies improve performance for short-duration stages?

- How generalizable is the model to different ages, strains, or staining protocols?

6. Conclusion

1. The conclusion is overly long and contains repetitive statements already addressed in the Discussion.

2. It should be condensed to focus on the principal findings and their implications.

3. Claims regarding “objective standards” should be tempered given the limited dataset size.

4. Questions

- Can the authors explicitly state the main scientific contribution and the main practical contribution of this study?

**********

6. PLOS authors have the option to publish the peer review history of their article (what does this mean?). If published, this will include your full peer review and any attached files.

If you choose “no”, your identity will remain anonymous but your review may still be made public.

**Do you want your identity to be public for this peer review?**  For information about this choice, including consent withdrawal, please see our Privacy Policy.

Reviewer #1: No

Reviewer #2: No

**********

[NOTE: If reviewer comments were submitted as an attachment file, they will be attached to this email and accessible via the submission site. Please log into your account, locate the manuscript record, and check for the action link "View Attachments". If this link does not appear, there are no attachment files.]

To ensure your figures meet our technical requirements, please review our figure guidelines: https://journals.plos.org/plosone/s/figures

You may also use PLOS’s free figure tool, NAAS, to help you prepare publication quality figures: https://journals.plos.org/plosone/s/figures#loc-tools-for-figure-preparation.

NAAS will assess whether your figures meet our technical requirements by comparing each figure against our figure specifications.

Attachment Submitted filename: Plos_one_review.docx

# Author comment: Author response to Decision Letter 1

**DOI:** 10.1371/journal.pone.0337245.r002

22 May 2026

PONE-D-25-59697

Automated spermatogenic staging in PAS-stained testes of Sprague-Dawley rats using a deep learning model for nomal and atrophied tissues

PLOS One

Dear Dr. Son,

Thank you for submitting your manuscript to PLOS ONE. After careful consideration, we feel that it has merit but does not fully meet PLOS ONE’s publication criteria as it currently stands. Therefore, we invite you to submit a revised version of the manuscript that addresses the points raised during the review process.

While it is an very interesting study, the robustness of the predictions shall be made basing on multiple observations from different slides of atrophied tissues.

We agree with your observation. The revised Discussion explicitly acknowledges that training included a limited number of atrophied specimens, including a single atrophied slide, and discusses the implications for generalizability. The need for validation using larger cohorts with multiple atrophied samples is now clearly stated as a major future direction.

Low precision score in predicting the Stage V should be justified and the possible technical reasons should be described thoroughly

Thank you for the remark. The Discussion now provides a detailed explanation that stage V exhibits broad morphological variability and substantial overlap with adjacent stages due to its wide acrosomal angle range. Technical challenges related to partial or oblique tubule sectioning are also discussed, consistent with known difficulties in manual staging.

Provide details on biological processes in which multi-class staging is carried out and justify its applicability and that it is better than the binary staging process.

We have added a dedicated explanation in the discussion, clarifying that spermatogenesis is a cyclic, stage-specific process and that toxicant effects can target specific stages. Multi-class staging enables localization of disruption along the spermatogenic continuum and provides greater mechanistic insight than binary (normal vs. atrophic) classification.

Provide complete technical details and restrict to the results obtained without overemphasising.

Technical details regarding model design, inference efficiency, and performance trade-offs are now described concisely and in direct relation to the reported results, avoiding overemphasis or speculative claims.

Strong rationale for carrying out this study and justification that is based on previous studies should be included in the introduction. Moreover, the superiority of the assessment made over the existing ones should also be thoroughly presented.

The Introduction has been revised to more clearly position this study relative to prior segmentation-based approaches, highlighting the advantages of an object detection–based framework combined with PAS staining in achieving full stage resolution with improved computational efficiency.

Discussion should be robust and avoid reiterating the observed results. Conclusions should focus on what are the benefits of this study in research and clinical terms and what are the future possibilities for the results presented in this study.

The Discussion has been rewritten to emphasize biological interpretation rather than result reiteration. The Conclusion now explicitly outlines research benefits, practical implications for toxicologic pathology, and future applications.

Please submit your revised manuscript by Mar 01 2026 11:59PM. If you will need more time than this to complete your revisions, please reply to this message or contact the journal office at plosone@plos.org. When you're ready to submit your revision, log on to https://www.editorialmanager.com/pone/ and select the 'Submissions Needing Revision' folder to locate your manuscript file.

Please include the following items when submitting your revised manuscript:

• A letter that responds to each point raised by the academic editor and reviewer(s). You should upload this letter as a separate file labeled 'Response to Reviewers'.

• A marked-up copy of your manuscript that highlights changes made to the original version. You should upload this as a separate file labeled 'Revised Manuscript with Track Changes'.

• An unmarked version of your revised paper without tracked changes. You should upload this as a separate file labeled 'Manuscript'.

If you would like to make changes to your financial disclosure, please include your updated statement in your cover letter. Guidelines for resubmitting your figure files are available below the reviewer comments at the end of this letter.

If applicable, we recommend that you deposit your laboratory protocols in protocols.io to enhance the reproducibility of your results. Protocols.io assigns your protocol its own identifier (DOI) so that it can be cited independently in the future. For instructions see: https://journals.plos.org/plosone/s/submission-guidelines#loc-laboratory-protocols. Additionally, PLOS ONE offers an option for publishing peer-reviewed Lab Protocol articles, which describe protocols hosted on protocols.io. Read more information on sharing protocols at https://plos.org/protocols?utm_medium=editorial-email&utm_source=authorletters&utm_campaign=protocols.

We look forward to receiving your revised manuscript.

Kind regards,

Suresh Yenugu

Academic Editor

PLOS One

Journal Requirements:

When submitting your revision, we need you to address these additional requirements.

1. Please ensure that your manuscript meets PLOS ONE's style requirements, including those for file naming. The PLOS ONE style templates can be found at

https://journals.plos.org/plosone/s/file?id=wjVg/PLOSOne_formatting_sample_main_body.pdf and

https://journals.plos.org/plosone/s/file?id=ba62/PLOSOne_formatting_sample_title_authors_affiliations.pdf

We have reviewed the manuscript and confirmed that it meets the PLOS ONE style requirements, including file naming conventions.

2. To comply with PLOS One submissions requirements, in your Methods section, please provide additional information regarding the experiments involving animals and ensure you have included details on (1) methods of sacrifice, (2) methods of anesthesia and/or analgesia, and (3) efforts to alleviate suffering.

The requested information regarding animal experiments, including methods of sacrifice, anesthesia/analgesia, and efforts to alleviate suffering, has been added to the Methods section.

3. Please note that your Data Availability Statement is currently missing the repository name. If your manuscript is accepted for publication, you will be asked to provide these details on a very short timeline. We therefore suggest that you provide this information now, though we will not hold up the peer review process if you are unable.

The repository name (Figshare) has been added to the Data Availability Statement.

4. Thank you for stating the following in the Competing Interests section:

[The authors have declared that no competing interests exist.].

We note that one or more of the authors are employed by a commercial company: CorestemChemon Nonclinical Research Institute

1. Please provide an amended Funding Statement declaring this commercial affiliation, as well as a statement regarding the Role of Funders in your study. If the funding organization did not play a role in the study design, data collection and analysis, decision to publish, or preparation of the manuscript and only provided financial support in the form of authors' salaries and/or research materials, please review your statements relating to the author contributions, and ensure you have specifically and accurately indicated the role(s) that these authors had in your study. You can update author roles in the Author Contributions section of the online submission form.

Please also include the following statement within your amended Funding Statement.

“The funder provided support in the form of salaries for authors [insert relevant initials], but did not have any additional role in the study design, data collection and analysis, decision to publish, or preparation of the manuscript. The specific roles of these authors are articulated in the ‘author contributions’ section.”

If your commercial affiliation did play a role in your study, please state and explain this role within your updated Funding Statement.

2. Please also provide an updated Competing Interests Statement declaring this commercial affiliation along with any other relevant declarations relating to employment, consultancy, patents, products in development, or marketed products, etc.

Within your Competing Interests Statement, please confirm that this commercial affiliation does not alter your adherence to all PLOS ONE policies on sharing data and materials by including the following statement: "This does not alter our adherence to PLOS ONE policies on sharing data and materials.” (as detailed online in our guide for authors http://journals.plos.org/plosone/s/competing-interests) . If this adherence statement is not accurate and there are restrictions on sharing of data and/or materials, please state these. Please note that we cannot proceed with consideration of your article until this information has been declared.

Please include both an updated Funding Statement and Competing Interests Statement in your cover letter. We will change the online submission form on your behalf.

The Funding Statement and Competing Interests Statement have been updated accordingly.

5. Please include your full ethics statement in the ‘Methods’ section of your manuscript file. In your statement, please include the full name of the IRB or ethics committee who approved or waived your study, as well as whether or not you obtained informed written or verbal consent. If consent was waived for your study, please include this information in your statement as well.

The full ethics statement has been added to the Methods section, including the name of the ethics committee and consent information.

6. Please upload a new copy of Figures 5, 8, 9, 10, S1, and S2 as the details are not clear. Please follow the link for more information: https://journals.plos.org/plosone/s/figures

To improve figure clarity while maintaining manageable file sizes, representative images were selected and partially enlarged to better display the relevant details.

7. Please ensure that you refer to Table S1 in your text as, if accepted, production will need this reference to link the reader to the table.

A reference to Table S1 has been added to the main text.

8. Please include captions for your Supporting Information files at the end of your manuscript (S4_table_old.pdf), and update any in-text citations to match accordingly. Please see our Supporting Information guidelines for more information: http://journals.plos.org/plosone/s/supporting-information.

The file S4_table_old.pdf was removed because it is no longer used.

If the reviewer comments include a recommendation to cite specific previously published works, please review and evaluate these publications to determine whether they are relevant and should be cited. There is no requirement to cite these works unless the editor has indicated otherwise.

[Note: HTML markup is below. Please do not edit.]

Reviewers' comments:

Reviewer's Responses to Questions

Comments to the Author

1. Is the manuscript technically sound, and do the data support the conclusions?

The manuscript must describe a technically sound piece of scientific research with data that supports the conclusions. Experiments must have been conducted rigorously, with appropriate controls, replication, and sample sizes. The conclusions must be drawn appropriately based on the data presented.

Reviewer #1: Partly

Reviewer #2: Partly

________________________________________

2. Has the statistical analysis been performed appropriately and rigorously?

Reviewer #1: Yes

Reviewer #2: Yes

________________________________________

3. Have the authors made all data underlying the findings in their manuscript fully available?

The PLOS Data policy requires authors to make all data underlying the findings described in their manuscript fully available without restriction, with rare exception (please refer to the Data Availability Statement in the manuscript PDF file). The data should be provided as part of the manuscript or its supporting information, or deposited to a public repository. For example, in addition to summary statistics, the data points behind means, medians and variance measures should be available. If there are restrictions on publicly sharing data—e.g. participant privacy or use of data from a third party—those must be specified.

Reviewer #1: Yes

Reviewer #2: Yes

________________________________________

4. Is the manuscript presented in an intelligible fashion and written in standard English?

PLOS ONE does not copyedit accepted manuscripts, so the language in submitted articles must be clear, correct, and unambiguous. Any typographical or grammatical errors should be corrected at revision, so please note any specific errors here.

Reviewer #1: Yes

Reviewer #2: No

________________________________________

5. Review Comments to the Author

Please use the space provided to explain your answers to the questions above. You may also include additional comments for the author, including concerns about dual publication, research ethics, or publication ethics. (Please upload your review as an attachment if it exceeds 20,000 characters)

Reviewer #1: The present study presents a region-based convolutional neural network designed to predict spermatogenic stages from PAS-stained testicular tissue sections. The authors have incorporated robust statistical procedures and report that the model can distinguish 14 stages with an average accuracy of 0.86. Additionally, the framework appears capable of quantitative grading by estimating proportional differences between atrophied and normal seminiferous tubules. Although the manuscript presents the data nicely, the placement of figure descriptions within the main text disrupts the logical flow of the manuscript. All figure legends should be positioned alongside the respective figures or compiled at the end of the discussion section.

Below are a few concerns regarding the model development and interpretation:

Major concerns

1. The manuscript does not sufficiently explain how the model mitigates overfitting or handles class imbalance. This issue is particularly relevant because the model was trained using images derived from only a single atrophic slide, raising concerns about the generalizability and robustness of the classifier.

In the Discussion, we have clarified how overfitting and class imbalance were addressed in the model, including the use of data augmentation applied across all stages and the intrinsic balancing effect of the Cascade R-CNN architecture. While the training data were derived from a single atrophic slide, the model was trained on a large number of morphologically diverse seminiferous tubules, which partially mitigates overfitting at the tubule level.

2. As shown in Figure 4, while most stages show satisfactory performance, the average precision for stage V remains below 0.7. The authors should provide a justification or discuss potential biological or technical reasons underlying this discrepancy.

This point is now addressed in detail in the Discussion. We explain that the lower AP for stage V reflects intrinsic biological variability and technical challenges in acrosomal angle assessment rather than model instability. This explanation is supported by established spermatogenic staging criteria and parallels known limitations in manual evaluation.

3. Given the substantial morphological similarity among spermatogenic stages, developing a multi-class classifier is inherently challenging. Are there any other existing image-based/histology-based models for a similar multi-class classification? The authors should also clarify how a multi-class stage prediction is a better approach than a preliminary binary classification (atrophy vs. normal).

We acknowledge your points. We explained in the Discussion why a multi-class prediction was chosen. Where a binary atrophy or normal classifier would detect gross abnormalities, o

Attachment Submitted filename: Response to Reviewers.docx

# Peer review: Decision Letter 1

**DOI:** 10.1371/journal.pone.0337245.r003

**Authors:** Suresh Yenugu, Suresh Yenugu

10 Jun 2026

Automated spermatogenic staging in periodic acid-Schiff-stained testes of Sprague–Dawley rats using a deep learning model for normal and atrophied tissues

PONE-D-25-59697R1

Dear Dr. Son,

We’re pleased to inform you that your manuscript has been judged scientifically suitable for publication and will be formally accepted for publication once it meets all outstanding technical requirements.

Within one week, you’ll receive an e-mail detailing the required amendments. When these have been addressed, you’ll receive a formal acceptance letter and your manuscript will be scheduled for publication.

An invoice will be generated when your article is formally accepted. Please note, if your institution has a publishing partnership with PLOS and your article meets the relevant criteria, all or part of your publication costs will be covered. Please make sure your user information is up-to-date by logging into Editorial Manager at Editorial Manager® and clicking the ‘Update My Information' link at the top of the page. For questions related to billing, please contact billing support.

If your institution or institutions have a press office, please notify them about your upcoming paper to help maximize its impact. If they’ll be preparing press materials, please inform our press team as soon as possible -- no later than 48 hours after receiving the formal acceptance. Your manuscript will remain under strict press embargo until 2 pm Eastern Time on the date of publication. For more information, please contact onepress@plos.org.

Kind regards,

Suresh Yenugu

Academic Editor

PLOS One

Additional Editor Comments (optional):

Reviewers' comments:

Reviewer's Responses to Questions

**Comments to the Author**

1. If the authors have adequately addressed your comments raised in a previous round of review and you feel that this manuscript is now acceptable for publication, you may indicate that here to bypass the “Comments to the Author” section, enter your conflict of interest statement in the “Confidential to Editor” section, and submit your "Accept" recommendation.

Reviewer #1: All comments have been addressed

Reviewer #2: All comments have been addressed

**********

2. Is the manuscript technically sound, and do the data support the conclusions?

The manuscript must describe a technically sound piece of scientific research with data that supports the conclusions. Experiments must have been conducted rigorously, with appropriate controls, replication, and sample sizes. The conclusions must be drawn appropriately based on the data presented.

Reviewer #1: Yes

Reviewer #2: Yes

**********

3. Has the statistical analysis been performed appropriately and rigorously?

Reviewer #1: Yes

Reviewer #2: Yes

**********

4. Have the authors made all data underlying the findings in their manuscript fully available?

The PLOS Data policy requires authors to make all data underlying the findings described in their manuscript fully available without restriction, with rare exception (please refer to the Data Availability Statement in the manuscript PDF file). The data should be provided as part of the manuscript or its supporting information, or deposited to a public repository. For example, in addition to summary statistics, the data points behind means, medians and variance measures should be available. If there are restrictions on publicly sharing data—e.g. participant privacy or use of data from a third party—those must be specified.

Reviewer #1: Yes

Reviewer #2: Yes

**********

5. Is the manuscript presented in an intelligible fashion and written in standard English?

PLOS ONE does not copyedit accepted manuscripts, so the language in submitted articles must be clear, correct, and unambiguous. Any typographical or grammatical errors should be corrected at revision, so please note any specific errors here.

Reviewer #1: Yes

Reviewer #2: Yes

**********

6. Review Comments to the Author

Please use the space provided to explain your answers to the questions above. You may also include additional comments for the author, including concerns about dual publication, research ethics, or publication ethics. (Please upload your review as an attachment if it exceeds 20,000 characters)

Reviewer #1: All the concerns are now addressed by the authors. Both major and minor points are discussed and included in the manuscript.

Reviewer #2: The authors have satisfactorily addressed all reviewer comments and revised the manuscript accordingly. The manuscript has improved substantially and is now suitable for publication

**********

7. PLOS authors have the option to publish the peer review history of their article (what does this mean?). If published, this will include your full peer review and any attached files.

If you choose “no”, your identity will remain anonymous but your review may still be made public.

**Do you want your identity to be public for this peer review?**  For information about this choice, including consent withdrawal, please see our Privacy Policy.

Reviewer #1: **Yes:** Manjari Kiran

Reviewer #2: No

**********

# Editor report: Acceptance letter

**DOI:** 10.1371/journal.pone.0337245.r004

**Authors:** Suresh Yenugu, Suresh Yenugu

PONE-D-25-59697R1

PLOS One

Dear Dr. Son,

I'm pleased to inform you that your manuscript has been deemed suitable for publication in PLOS One. Congratulations! Your manuscript is now being handed over to our production team.

At this stage, our production department will prepare your paper for publication. This includes ensuring the following:

* All references, tables, and figures are properly cited

* All relevant supporting information is included in the manuscript submission,

* There are no issues that prevent the paper from being properly typeset

You will receive further instructions from the production team, including instructions on how to review your proof when it is ready. Please keep in mind that we are working through a large volume of accepted articles, so please give us a few days to review your paper and let you know the next and final steps.

Lastly, if your institution or institutions have a press office, please let them know about your upcoming paper now to help maximize its impact. If they'll be preparing press materials, please inform our press team within the next 48 hours. Your manuscript will remain under strict press embargo until 2 pm Eastern Time on the date of publication. For more information, please contact onepress@plos.org.

You will receive an invoice from PLOS for your publication fee after your manuscript has reached the completed accept phase. If you receive an email requesting payment before acceptance or for any other service, this may be a phishing scheme. Learn how to identify phishing emails and protect your accounts at https://explore.plos.org/phishing.

If we can help with anything else, please email us at customercare@plos.org.

Thank you for submitting your work to PLOS ONE and supporting open access.

Kind regards,

PLOS ONE Editorial Office Staff

on behalf of

Dr. Suresh Yenugu

Academic Editor

PLOS One
